# Humanizing acidic mammalian chitinase variants establish lung immune conditioning and control environmentally driven inflammation and fibrosis

**DOI:** 10.1016/j.celrep.2026.117833

**Published:** 2026-08-12

**Authors:** Yilin Wang, Haerin Jung, Do-Hyun Kim, Eilene Liu, Donovan Watza, Samuel I. Risma, Kira L. Florczak, Leah M. Kim, Zeena Bou Reslan, Ananya Gupta, Ruppal Soni, Roberto Efraín Díaz, Richard M. Locksley, James S. Fraser, Janet S. Lee, Steven J. Van Dyken

**Affiliations:** 1 Department of Pathology & Immunology, Washington University School of Medicine, St. Louis, MO, USA; 2 Department of Life Science, College of Natural Sciences, Hanyang University, Seoul 04763, Republic of Korea; 3 Research Institute for Natural Sciences, Hanyang University, Seoul, Republic of Korea; 4 Research Institute for Convergence of Basic Sciences, Hanyang University, Seoul 04763, Republic of Korea; 5 Division of Pulmonary and Critical Care Medicine, Department of Medicine, Washington University School of Medicine, St. Louis, MO, USA; 6 Department of Bioengineering and Therapeutic Sciences, University of California, San Francisco, San Francisco, CA, USA; 7 Department of Microbiology & Immunology, University of California, San Francisco, San Francisco, CA, USA; 8 Department of Medicine, University of California, San Francisco, San Francisco, CA, USA; 9 Howard Hughes Medical Institute, University of California, San Francisco, San Francisco, CA, USA; 10 Present address: Department of Translational Immunology, Genentech, Inc., 1 DNA Way, South San Francisco, CA, USA; 11 Senior author; 12 Lead contact

## Abstract

Chitin, a widespread environmental particle constituent, triggers lung inflammation but is degraded by chitinases. In humans, single-nucleotide polymorphisms (SNPs) in ***CHIA*** (acidic mammalian chitinase; AMCase) are associated with lung disease, suggesting that chitinase variants influence responses to airborne particles. Here, we edit the mouse ***Chia1*** locus to generate humanized (hChia) mice harboring common human SNPs. Compared with controls expressing disease-protective SNPs, hChia mice lack robust chitinase activity and fail to degrade natural chitin substrates. Lung-resident lymphocytes and macrophages are spontaneously primed and sensitive to inflammatory triggering by environmental chitin. Immune cell infiltration correlates with airway chitin following challenge, and hChia mice exhibit exacerbated inflammatory and fibrotic lung disease. In humans with acute respiratory failure, alveolar hemorrhage coincides with environmentally derived chitin particles that are susceptible to chitinase degradation, attenuating inflammatory cell responses. Thus, environmental chitin and chitinase activity are crucial determinants of lung immune conditioning with potential therapeutic applications.

## In brief

Environmental chitin is continuously inhaled, yet the impact of common human AMCase variants on its turnover and inflammatory effects in the lung is unknown. Wang et al. demonstrate that low-activity AMCase variants condition lung immunity and promote exaggerated inflammatory responses to allergen exposure and fibrotic challenge.

## Graphical Abstract

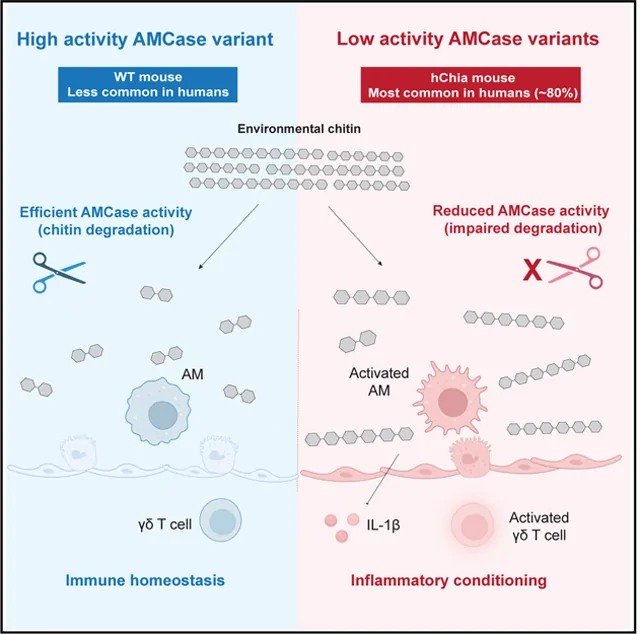

## INTRODUCTION

Airborne particles increase lung disease mortality risk and contribute to the global disease burden.^1–5^ Gene-environment interactions that govern lung inflammatory tone and mediate responses to inhaled particles, however, remain unclear. Chitin (poly-N-acetylglucosamine) is ubiquitous in the environment as a structural component of arthropods and fungi but can be degraded by equally widespread chitinases expressed by bacteria, plants, and animals that exhibit evolutionarily conserved enzymatic function.^6–10^ Mammals express endo-chitinases (EC 3.2.1.14) chitotriosidase (Chit1) and AMCase (Chia), which catalyze internal cleavage of chitin polysaccharide chains; however, these enzymatic activities have largely been established using synthetic N-acetylglucosamine (GlcNAc) oligomeric substrates. In contrast, naturally occurring chitin particles are highly insoluble crystalline scaffolds, often mineralized and complexed with an array of immunostimulatory proteins, lipids, and carbohydrates derived from invertebrate hosts. Recalcitrant chitin particles are widely distributed in human and mouse environments and have been implicated as drivers of inflammatory and fibrotic lung disease.^8,10^

AMCase is produced in the respiratory tract by epithelial secretory cells and can bind and degrade chitin,^8,10^ but it has remained unclear how common human AMCase enzyme activity variants impact the turnover of naturally occurring chitin particles and associated inflammatory responses *in vivo*. Human and mouse AMCase sequences share 81% identity.^11^ Three genetically linked nonsynonymous single-nucleotide point mutations (single-nucleotide polymorphisms [SNPs]) are found in the most common human *CHIA* haplotypes (G290A, A296G, and T339G, resulting in amino acid alterations D45N, N47D, and M61R, respectively).^12^ Most humans express the NDR-containing AMCase isoform, which exhibits significantly reduced chitinase activity compared to the less-common isoform containing DNM residues identical to those present in wild-type mouse AMCase and is associated with protection from inflammatory lung disease in humans.^12–14^ However, the physiologic role of these human *CHIA* SNPs in mediating AMCase enzymatic function in relation to naturally occurring chitin particles and their influence on environmentally driven inflammatory lung disease have not been established *in vivo*. Here, using gene-editing approaches, we generate mice harboring these key SNPs to uncover how common human AMCase variants control an environmentally driven process for conditioning lung-resident immune cells that impacts inflammatory and fibrotic lung disease outcomes.

## RESULTS

### Altered chitinase activity and chitin degradation in humanized AMCase mice

**Figure 1.**
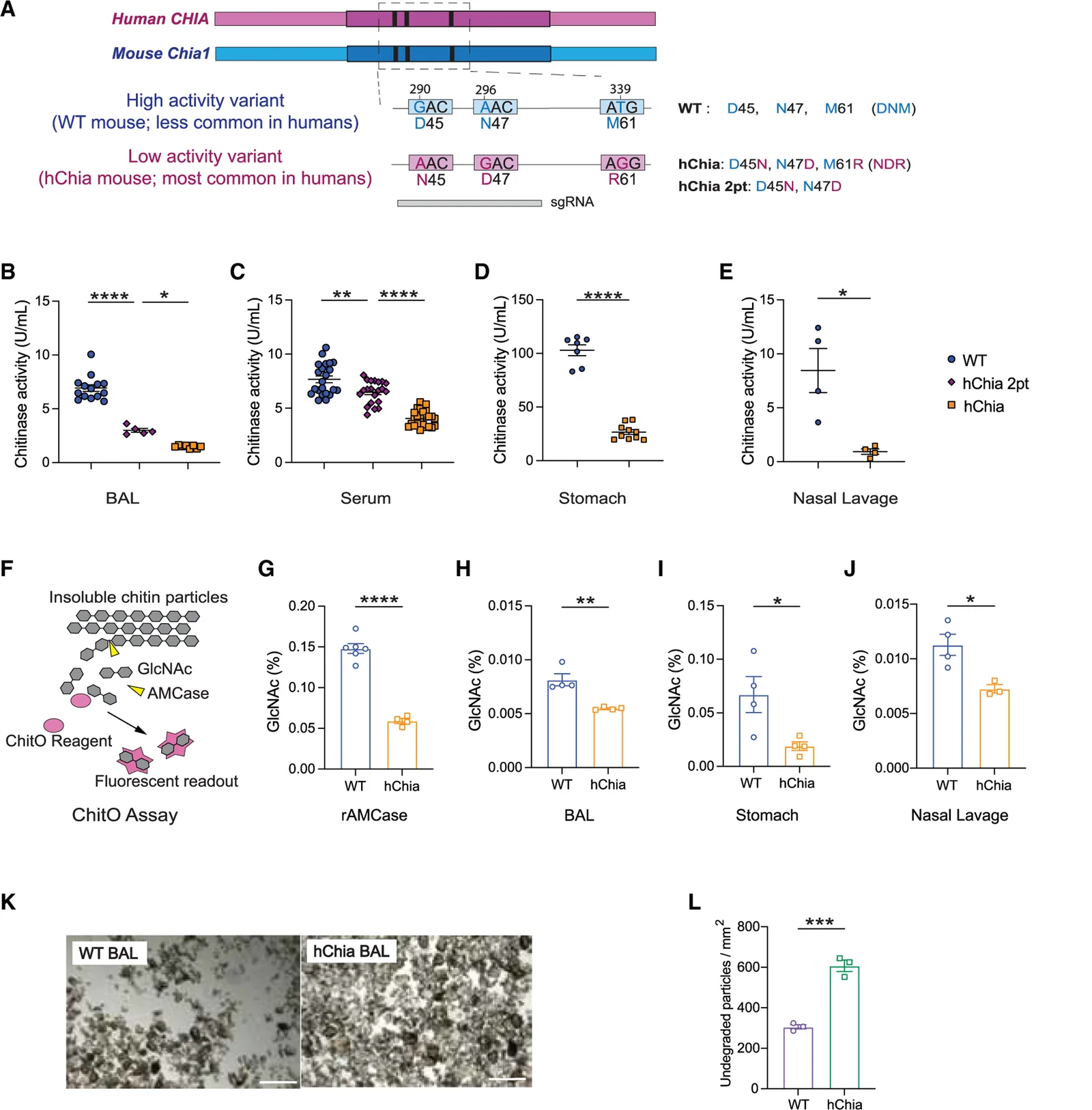
Altered chitinase activity in humanized AMCase mice (A) CRISPR-Cas9 gene editing strategy to generate 2-point and 3-point humanized Chia1 (hChia) alleles using a single-guide RNA (sgRNA) and donor ssODN (single-stranded oligodeoxynucleotide). (B–E) Chitinase activity in bronchoalveolar lavage (BAL; B), serum (C), stomach lavage (D), and nasal lavage (E) isolated from wild-type (WT), hChia, and hChia-2pt mice. BAL, stomach lavage, and nasal lavage chitinase activity were normalized to total protein amounts. (F) Monitoring of chitin particle degradation and N-acetylglucosamine (GlcNAc) production using ChitO assay. Part of the illustration was created with Biorender.com. (G–J) ChitO assay-based quantification of GlcNAc release from purified chitin particles using recombinant WT and hChia AMCase variants (G), or BAL (H), stomach lavage (I), and nasal lavage (J) collected from WT or hChia mice. (K and L) Representative images (K) and quantification (L) of chitin particle degradation by BAL from WT and hChia mice. Scale bars, 250 μm. Data are presented as mean ± SEM and are pooled from at least 2 independent experiments; individual data points represent biological replicates. Statistical analyses were performed using unpaired *t* test (D, E, G–J, and L) or one-way ANOVA (B and C). *p < 0.05, **p < 0.01, ***p < 0.001, ****p < 0.0001.

To study AMCase activity variants in homeostasis and disease settings, we generated humanized “hChia” mice by genetically editing the endogenous mouse AMCase (*Chia1*) locus to incorporate common human SNPs. We generated two separate founder mouse lines, one with two SNPs (hChia-2pt; G290A/A296G resulting in D45N/N47D), and one with three SNPs (hChia; G290A/A296G/T339G, resulting in D45N/N47D/M61R) representing the trio of linked SNPs present in the most common human haplotypes (Figure 1A).^12^ Compared to variants with the wild-type (WT; mouse-like) SNPs, endochitinase (i.e., chitobiosidase) activity was diminished in bronchoalveolar lavage (BAL) fluid and serum from both double (hChia-2pt) and triple (hChia) humanizing AMCase mice, with more significant reductions in hChia mice harboring all three mutations (Figures 1B and 1C), corroborating results from recombinantly expressed AMCase isoforms.^12–14^ We confirmed that *Chia1* mRNA expression in lung tissue and AMCase protein amounts in the BAL fluid (normalized to total protein) were comparable between WT and hChia mice (Figures S1A–S1C), suggesting that differences in basal AMCase expression did not account for the marked reduction in chitinase activity observed in hChia mice. Chitinase activity was also nearly absent in the stomach and nasal lavage from hChia mice compared to WT controls (Figures 1D and 1E), establishing that these three residues in the AMCase catalytic domain influence endochitinase activity in multiple tissues *in vivo* and providing mouse tools to study the physiologic effects of common human AMCase variants.

Since chitin forms an insoluble structural component of naturally occurring particles, we tested the effects of hChia mutations on chitinase degradation of crystalline chitin substrates. We quantified degradation of insoluble chitin particles in the presence of mucosal fluids collected from WT and hChia mice or with recombinant AMCase isoforms, while monitoring the release of GlcNAc oligomer reaction products into the supernatant using a previously described chito-oligosaccharide oxidase (ChitO) assay (Figure 1F).^14–16^ Upon incubation with chitin particles, BAL, stomach lavage, and nasal lavage from hChia mice showed reduced GlcNAc production compared to WT controls, in agreement with observations using recombinant WT and hChia protein isoforms and reflecting diminished chitinase activity on insoluble crystalline chitin substrates *in vitro* and *in vivo* (Figures 1G–1J). Accordingly, chitin particle degradation, assessed by microscopy, was impaired in hChia BAL fluid compared to WT control (Figures 1K and 1L), indicating that humanizing AMCase mutations impact the degradation of natural chitin substrates by reducing the overall capacity of lung secretions to enzymatically process chitin particles.

### Humanized AMCase mice exhibit environmentally driven lung inflammatory conditioning

**Figure 2.**
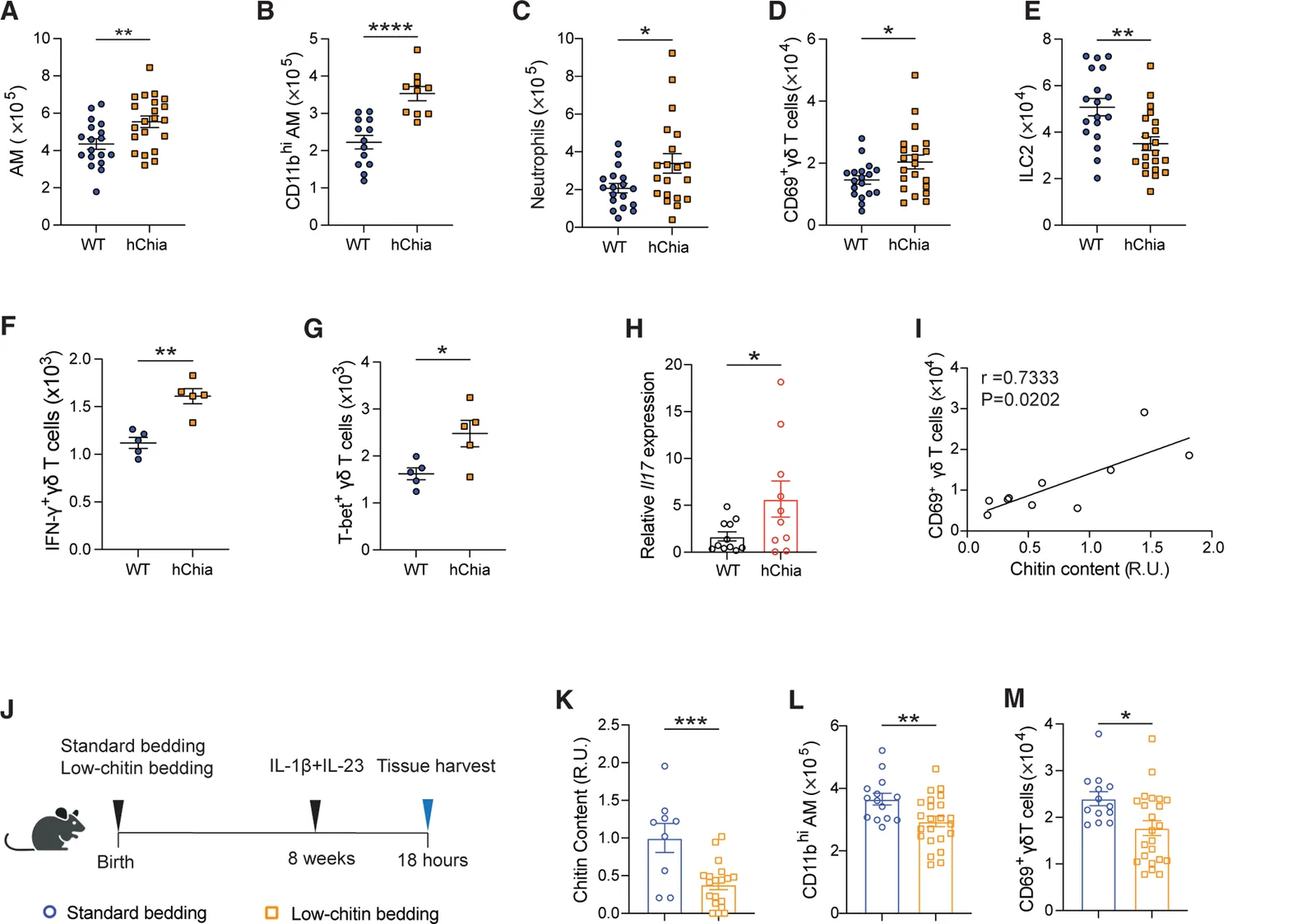
Environmentally driven lung inflammatory conditioning in hChia mice (A–E) Flow cytometric quantification of alveolar macrophages (AMs; A), CD11bhi AMs (B), neutrophils (C), activated CD69^+^ γδ T cells (D), and group 2 innate lymphoid cells (ILC2s; E) in lung tissue from WT and hChia mice at steady state. (F and G) Quantification of IFNγ^+^ and T-bet^+^ γδ T cells in lung tissue. (H) Relative expression of Il17 in whole lung homogenates measured by qPCR. (I) Relationship between BAL chitin content and CD69^+^ γδ T cell numbers in individual mice. (J–M) Humanized (hChia) mice were housed on standard or low-chitin bedding from birth and challenged intranasally with IL-1β + IL-23 at 8 weeks of age (J). Chitin content in BAL (K), lung CD11bhi AMs (L), and activated CD69^+^ γδ T cells (M) in the lungs of hChia mice 18 h after cytokine stimulation. Data are presented as mean ± SEM and are pooled from at least 2 independent experiments; individual data points represent biological replicates. Statistical analyses were performed using unpaired *t* test (A–H and K–M) or Spearman correlation (I). *p < 0.05, **p < 0.01, ***p < 0.001, ****p < 0.0001.

In mice, environmental chitin particles can activate lung-resident lymphocytes to drive inflammatory cell recruitment, which is AMCase dependent in standard mouse caging conditions.^8,10^ We examined the effect of humanizing AMCase variants on environmental chitin substrate accumulation in the airways of 8- to 12-week-old hChia and WT mice by assaying chitin amounts in the BAL fluid. Although we did not detect statistically significant differences between genotypes, we noted that airway chitin amounts were highly variable between individual mice (Figure S3A), consistent with prior observations in AMCase knockout (KO) mice^8^ and supporting the notion that environmental chitin in standard mouse caging conditions may influence lung immune priming processes. Additionally, recombinant WT AMCase protein was more effective at degrading naturally occurring environmental chitin particles recovered from mouse airways than the hChia AMCase protein isoform (Figure S3B), suggesting a dynamic interplay between inhaled particles, chitinase activity, and lung-resident immune cells. Indeed, numbers of alveolar macrophages (AMs) were significantly increased in the lungs of hChia compared to WT mice in the steady state and exhibited a CD11b^hi^ phenotype indicative of priming and acquisition of a pro-fibrotic state (Figures 2A and 2B).^17^ Activated (CD69^+^) innate-like γδ T cells and neutrophils were also elevated in the lungs of hChia mice compared to controls (Figures 2C and 2D), resembling infiltrates induced by inhaled chitin particles^18–21^ and consistent with environmentally driven accumulation of these cells. Although a modest increase in steady-state lung eosinophils was noted in hChia mice compared to controls, this difference was not statistically significant (Figure S3C), and lung group 2 innate lymphoid cells (ILC2s) were reduced in the lungs of hChia mice compared to controls (Figure 2E), contrasting with prior observations in AMCase KO mice^8,10^ and suggesting that AMCase activity variants differentially shape lung inflammatory tone by modulating resident immune cell priming.

Consistent with this, DC1 populations, IFNγ^+^ γδ T cells, and T-bet^+^ γδ T cell subsets were significantly increased in the lungs of hChia compared to control mice at steady state (Figures 2F, 2G, S3D, and S3E). Although numbers of other cytokine-producing T cell subsets (i.e., IL-4^+^CD4^+^, IL-17^+^CD4^+^, IFNγ^+^CD4^+^ T cells, and IL-17^+^ γδ T cells) and lung *Il1b* and *Il13* expression were not altered (Figures S3F–S3K), *Il17* mRNA expression was significantly increased in the lungs of hChia compared to control mice (Figure 2H), consistent with increased numbers of activated γδ T cells. Because γδ T cells represent a key cellular target of chitin-induced lung inflammation,^8,20^ we next examined relationships between airway chitin burden and innate-like T cell activation. Indeed, environmental chitin amounts in the airways significantly correlated with numbers of activated γδ T cells in lung tissue (Figure 2I), while immune cells in BAL fluid as well as lung histology were relatively unaltered in hChia versus control mice (Figure S4), suggesting that sustained exposure to environmental chitin conditions tissue-resident innate and innate-like immune cells in the lungs.

We next tested whether AM priming and γδ T cell activation in the lungs of hChia mice could be attenuated by reducing environmental chitin exposure. We reared hChia mice on low-chitin or standard bedding from birth and then challenged them intranasally with the inflammatory cytokines IL-1β and IL-23 to trigger robust γδ T cell responses (Figure 2J). Compared to standard housing conditions, low-chitin conditions reduced the chitin content in the airways of adult hChia mice, which also exhibited attenuated AM and γδ T cell responses to IL-1β and IL-23 stimulation (Figures 2K–2M). These data suggest that in the steady state, humanizing AMCase activity variants differentially process naturally occurring environmental particles *in vivo*, thereby conditioning the lung-resident AM and lymphocyte populations to establish tonic responsiveness to local inflammatory cues.

To determine whether these effects were restricted to the lung, we also examined systemic immune and metabolic parameters in hChia mice. Body weight, fat mass, gastrointestinal eosinophils, and ILC2s were unaltered in hChia compared to control mice (Figures S5A–S5F). DC1 populations were elevated in the spleens of hChia mice compared to controls, consistent with their increased numbers in the lung, whereas splenic DC2, CD4^+^ T cells, and γδ T cells were comparable (Figures S5G–S5L), indicating that local lung immune activation was primarily due to environmental chitin accumulation rather than alterations in systemic metabolism or T cell homeostasis.

Because ILC2s were reduced in hChia lungs, we additionally tested whether cytokine-driven type 2 responses remained intact in these mice. Compared to phosphate-buffered saline (PBS)-treated controls, intranasal TSLP (thymic stromal lymphopoietin) + IL-33 treatment robustly induced eosinophil accumulation, ILC2 expansion, and T cell activation in the lungs of hChia and control mice (Figure S6A), confirming the functionality of ILC2s and other lung-resident cells in response to these activating cytokines. The magnitude of lung immune cell expansion was also comparable in both genotypes (Figures S6B–S6H), suggesting that high doses of activating cytokines can overcome the effects of steady-state conditioning by environmental particles. Together, these findings support a role for environmental chitin and AMCase activity variants in conditioning lung-resident immune populations in the steady state.

### AMCase activity variants influence inflammatory lung disease

**Figure 3.**
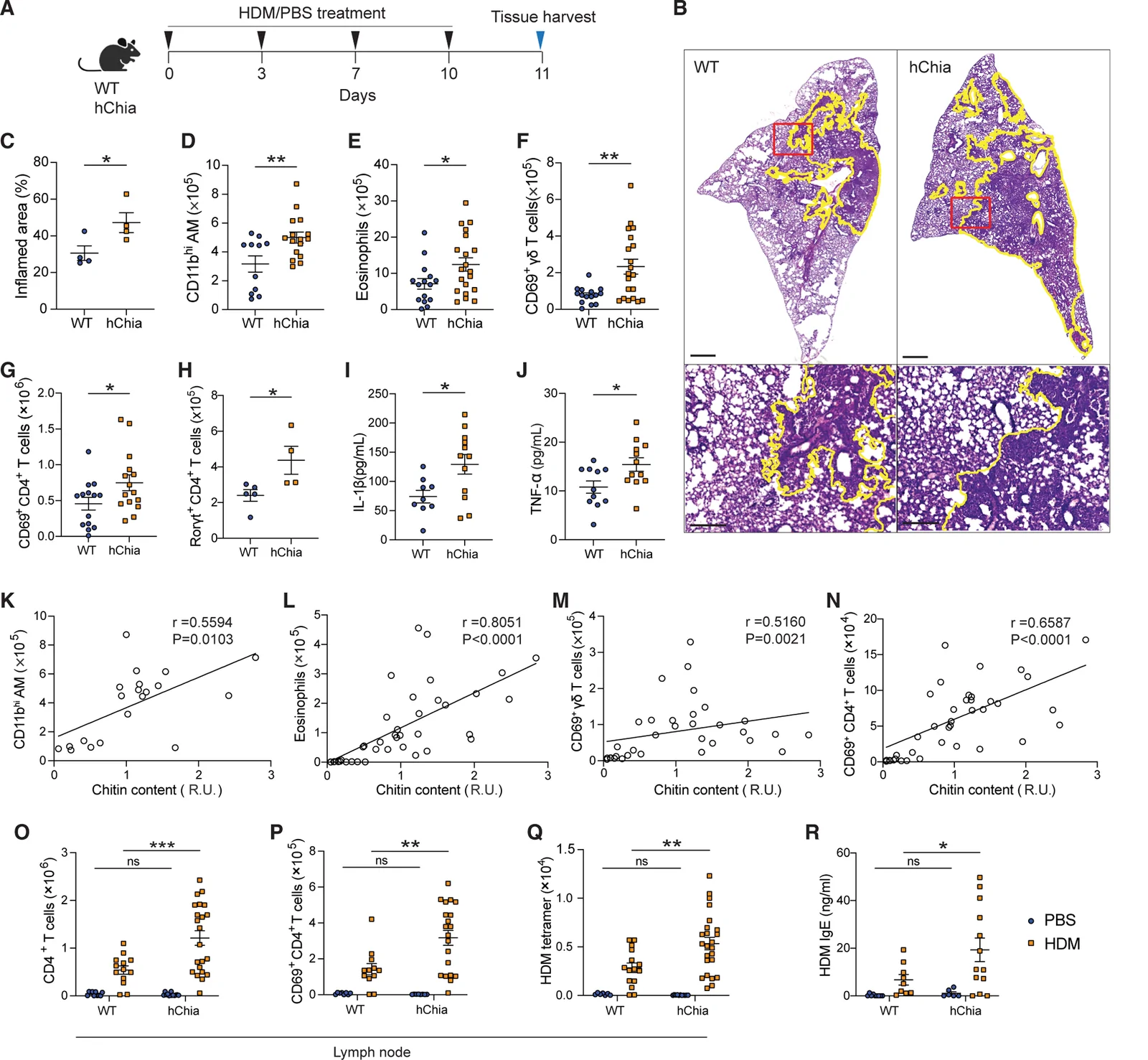
AMCase activity variants influence inflammatory lung disease (A) House dust mite (HDM) allergic airway challenge in WT and hChia mice. (B and C) Representative H&E-stained lung sections (B) and quantification of inflamed regions (C) from HDM-challenged mice. Inflamed regions are outlined in yellow. Scale bars, 1 mm (top); 250 μm (bottom). (D–J) Quantification of CD11bhi alveolar macrophages (AMs) (D), eosinophils (E), CD69^+^γδ T cells (F), CD69^+^CD4^+^ T cells (G), and RORγt^+^ CD4^+^ T cells (H) in lung tissue, and IL-1β (I) and TNF-α (J) concentrations in BAL fluid following HDM challenge. (K–N) Correlations between BAL chitin and CD11bhi AMs (K), eosinophils (L), CD69^+^γδ T cells (M), and CD69^+^CD4^+^ T cells (N) in the lungs of HDM-treated mice. (O–R) Total CD4^+^ T cells (O), CD69^+^CD4^+^ T cells (P), and HDM-specific CD4^+^ T cells (Q) in draining lymph nodes, and HDM-specific serum IgE (R) in WT and hChia mice following HDM challenge. Data are presented as mean ± SEM and are pooled from at least 2 independent experiments; individual data points represent biological replicates. Statistical analyses were performed using unpaired *t* test (C–J), Spearman correlation (K–N), or two-way ANOVA with Sidak’s multiple-comparison test (O–R). *p < 0.05; **p < 0.01; ***p < 0.001; ns, not statistically significant.

Since the three linked SNPs in hChia mice are associated with asthma susceptibility in humans,^12^ we next asked whether the elevated inflammatory set point in the lungs of humanized AMCase variant mice influenced immune responses to respiratory allergen challenge. We intranasally administered house dust mite (HDM) particles (minimally processed to retain natural chitin^8,21^) or PBS to WT and hChia mice (Figure 3A). As expected, HDM challenge induced lung inflammatory responses in all mice; however, the lungs of hChia mice were more extensively inflamed, marked by elevated lung CD11b^hi^ AMs, eosinophils, activated γδ T cells, and increased BAL IL-1β and TNF-α compared to WT controls (Figures 3B–3F, 3I, and 3J); BAL IL-5, IL-13, IL-17, and IL-6 were similar (Figures S7A–S7D). Airway chitin amounts correlated with lung inflammatory cells including CD11b^hi^ AMs, eosinophils, and activated γδ T cells (Figures 3K–3M), indicating that chitin acts as a natural adjuvant in the context of allergic lung inflammation. Consistent with this, we also observed that airway chitin correlated with activated CD4^+^ T cells in lung tissue, and both activated CD4^+^ T cells and RORγt-expressing T helper 17 (Th17) cells were elevated in the lungs of hChia compared to WT mice (Figures 3G, 3H, and 3N), suggesting that adaptive immune responses to HDM were shaped by humanizing AMCase variants. Indeed, we observed elevated numbers of CD4^+^ T cells, including activated and HDM-specific CD4^+^ T cells in lung-draining lymph nodes, as well as increased HDM-specific serum IgE in hChia compared to control mice following HDM allergen exposure (Figures 3O–3R).

As respiratory viral disease can be exacerbated by environmental particles and has also recently been linked to epithelial AMCase,^10^ we tested how AMCase variants influenced lung inflammatory responses to influenza A virus (IAV; Figure S8A). WT and hChia mice exhibited comparable body weight changes following IAV inoculation, and all mice survived (Figure S8B), contrasting with IAV-induced mortality in AMCase KO mice^10^ and indicating that anti-viral responses were intact in hChia mice. However, lung eosinophils and alveolar epithelial progenitor-like cells (AEPs), cell types influenced by environmental chitin,^10^ were increased in the lungs of hChia compared to WT mice at 21 days post-infection (dpi) with IAV (Figures S8C–S8D), suggesting that lung immune function and epithelial turnover were altered in hChia mice following respiratory viral infection. To model an allergic inflammatory exacerbation in the setting of post-viral lung disease, we then examined immune responses in IAV-infected WT and hChia mice that were additionally challenged with HDM at 34 and 36 dpi IAV (Figure S8A). Compared to IAV+HDM-exposed WT controls, lungs from hChia mice exhibited increased numbers of eosinophils, activated CD4^+^ T cells, and γδ T cells, accompanied by elevated IL-1β levels in BAL fluid and enlarged areas of inflamed lung tissue (Figures S8E–S8K), consistent with increased sensitivity to environmentally driven inflammatory triggering in hChia mice and supporting a role for AMCase activity variants in controlling post-viral inflammatory disease exacerbations.

### Pulmonary fibrosis is exacerbated in humanized hChia mice

**Figure 4.**
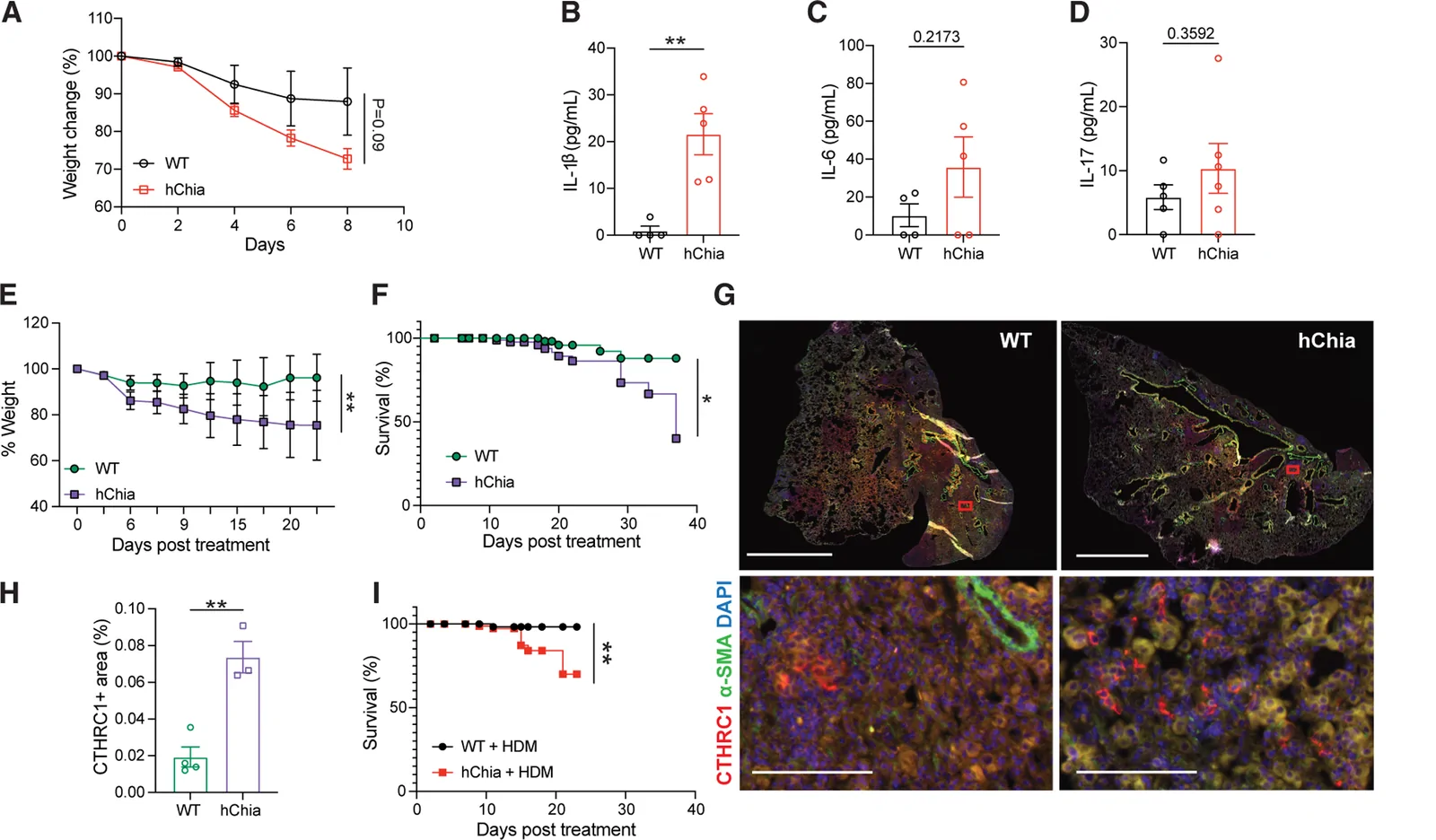
Pulmonary fibrosis is exacerbated in humanized hChia mice (A) Body weight change in WT and hChia mice following bleomycin treatment over time (WT, *n* = 4; hChia, *n* = 5). (B–D) BAL fluid cytokine measurements of IL-1β (B), IL-6 (C), and IL-17 (D) at day 8 after bleomycin treatment. (E and F) Body weight (E) and survival (F) of WT and hChia mice following bleomycin (WT, *n* = 9; hChia, *n* = 12). (G and H) Representative lung tissue staining (G) and quantification (H) of CTHRC1^+^ cells (red, CTHRC1; green, α-SMA; blue, DAPI) per tissue area in WT and hChia mice 42 days after bleomycin treatment. Scale bars, 2 mm (top); 100 μm (bottom). (I) Survival of WT and hChia mice challenged with HDM following bleomycin injury (I), as described in the STAR Methods (WT + HDM, *n* = 9; hChia + HDM, *n* = 14). Data are presented as mean ± SEM and are pooled from at least 2 independent experiments; individual data points represent biological replicates. Statistical analyses were performed using unpaired *t* test (B–D and H), mixed-effects model (restricted maximum likelihood (REML)) for body weight changes over time (A and E) or log rank (Mantel-Cox) test (F and I). *p < 0.05; **p < 0.01; ***p < 0.001.

Since hChia mice consistently displayed elevated IL-1β and γδ T cell signatures implicated in lung fibrosis and chitin responses,^8,20,22–24^ we tested how humanizing AMCase mutations impacted the course of pulmonary fibrosis using a model of intranasal bleomycin administration, which injures AMCase-producing epithelial cells^10^ and induces fibroblastic remodeling that largely resolves in WT mice over time. We noted a small but not statistically significant divergence in the body weights of hChia compared to WT mice 8 days after bleomycin exposure (Figure 4A). Although lung immune cells did not differ at this time point, hChia mice exhibited markedly elevated levels of the profibrotic inflammatory cytokine IL-1β in BAL fluid compared to WT controls (Figure 4B), while the levels of other fibrosis-associated cytokines IL-6 and IL-17 were unaltered (Figures 4C and 4D).

At later time points following bleomycin exposure, hChia mice exhibited significantly greater weight loss and reduced survival in comparison to WT controls (Figures 4E and 4F). Among surviving hChia mice, lung tissue showed more persistent scarring, including more extensive fibrotic lesions 42 days after bleomycin challenge, which included greater numbers of cells expressing the profibrotic myofibroblast marker CTHRC1 compared to controls (Figures 4G and 4H),^24^ indicating that the severity and persistence of pulmonary fibrosis are worsened by inefficient clearance of environmental inflammatory particles in low-activity humanized AMCase variant mice. Consistent with this idea, intranasal administration of environmental chitin-containing HDM particles during the initial inflammatory phase after bleomycin injury accelerated the mortality of hChia mice compared to WT controls (Figure 4I), suggesting that impaired chitin degradative capacity increases vulnerability to severe outcomes driven by inflammatory particulates after lung epithelial injury. Thus, across mouse models of acute and chronic lung disease, humanizing AMCase activity variants control IL-1β-associated inflammation and immune responses to multiple exacerbating environmental agents, as well as epithelial injury-induced tissue remodeling, implicating a shared mechanism by which environmental chitin particles promote persistent inflammatory and fibrotic signaling.

### Chitinase reduces mechanosensitive inflammatory effects of chitin from humans with acute lung injury

**Figure 5.**
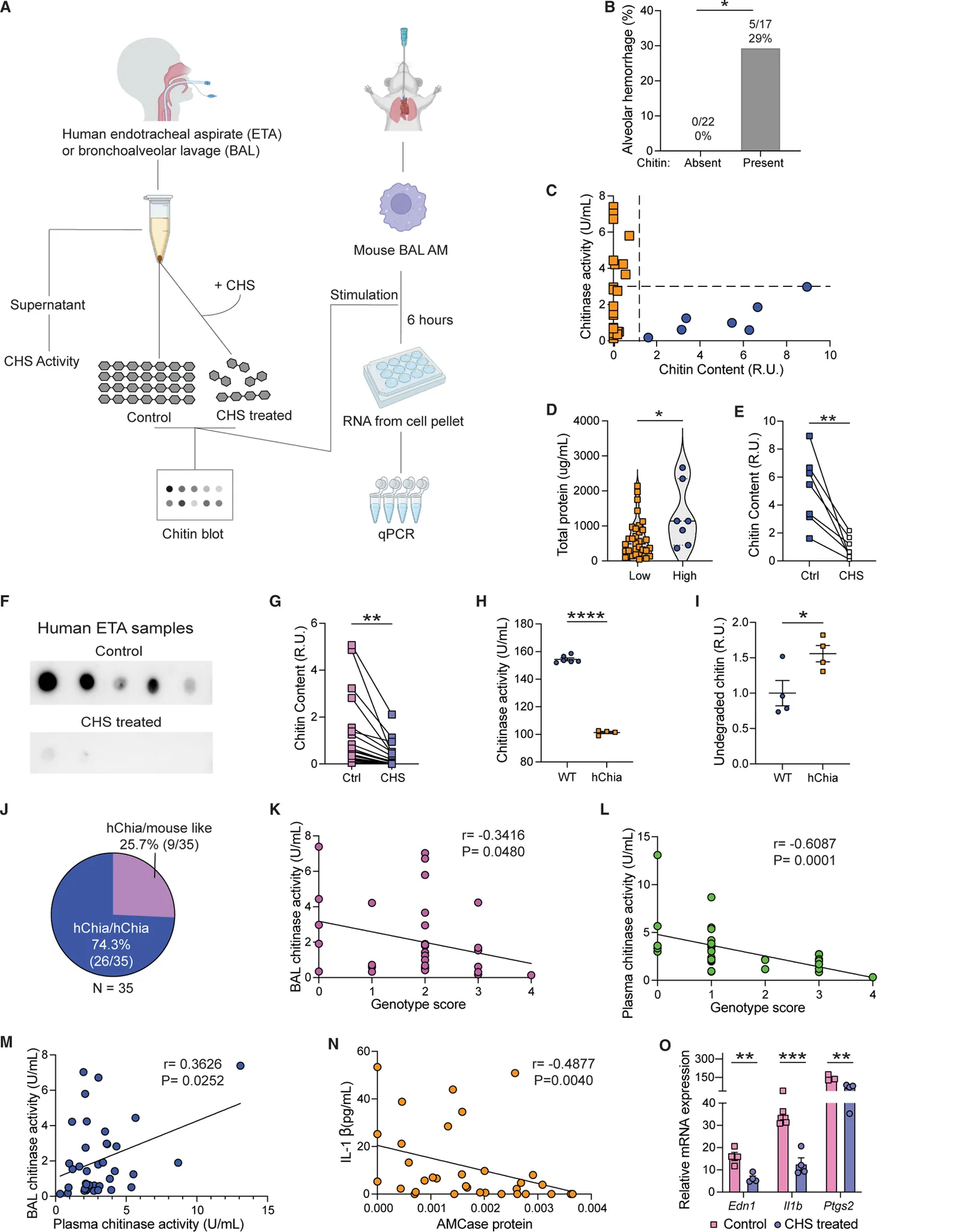
Inflammatory chitin from humans with acute lung injury is degraded by chitinases (A) Human endotracheal aspirates (ETAs) or bronchoalveolar lavage (BAL) fluid was screened for chitinase activity in the supernatant and chitin content in pellet fraction. Control- or chitinase (CHS)-treated chitin particles from human BAL pellet fractions were incubated with mouse alveolar macrophages (AMs) for qPCR analysis. Part of the illustration was created with Biorender.com. (B) Frequency of alveolar hemorrhage in BAL samples containing chitin (present) or samples with undetectable chitin (absent). (C) Chitinase activity and chitin content in BAL samples. (D) Total protein concentrations in chitin-low and chitin-high BAL samples. (E) Quantification of BAL chitin before (ctrl) and after chitinase (CHS) treatment. Lines indicate paired samples. (F) Representative chitin blot of control- and CHS-treated ETA samples. (G) Quantification of chitin levels in human ETAs before (ctrl) and after chitinase (CHS) treatment. Lines connect paired samples. (H and I) Chitinase activity of recombinant WT and hChia AMCase variants as assessed on soluble chitin substrates (H) and environmental chitin particles recovered from human ETA samples (I; expressed at the amount of undegraded chitin following incubation with indicated variant). (J) Distribution of CHIA genotypes among patient BAL samples. Pie chart indicates the proportion of individuals homozygous for hChia-associated SNPs (G290A, A296G, T339G; CHIAhChia/hChia) or heterozygous for hChia-associated SNPs and mouse-like (A290G, G296A, G339T) SNPs (CHIAhChia/mouse-like). (K) Relationship between composite chitinase genotype score and BAL fluid chitinase activity. Composite genotype scores were assigned based on combined CHIA (hChia or mouse-like SNPs) and CHIT1 (WT, CHIT1WT or 24-bp duplication, CHIT1dup) genotypes, as follows: CHIAhChia/mouse-like/CHIT1WT/WT = 0, CHIAhChia/hChia/CHIT1WT/WT = 1, CHIAhChia/mouse-like/CHIT1WT/dup = 2, CHIAhChia/hChia/CHIT1WT/dup = 3, and CHIAhChia/hChia/CHIT1dup/dup = 4. Also see Table S2. (L) Correlation between composite chitinase genotype score and plasma chitinase activity. (M) Correlation between BAL fluid and plasma chitinase activity. (N) Correlation between AMCase and IL-1β protein levels in human airway samples. AMCase protein abundance was normalized to total BAL protein. (O) Induction of inflammatory genes (Il1b, Edn1, and Ptgs2) in mouse AMs stimulated for 6 h with control- or CHS-treated human ETAs as shown in (A), normalized to untreated controls. Data are presented as mean ± SEM and are pooled from at least 2 independent experiments; individual data points represent biological replicates. Statistical analyses were performed using two-tailed Fisher’s exact test (B), unpaired *t* test (D, H–I, and O), paired *t* test (E–G), or Spearman correlation analysis (K–N). *p < 0.05, **p < 0.01, ***p < 0.001, ****p < 0.0001.

To relate these findings to human disease, we measured environmental chitin and chitinase activity in endotracheal aspirates (ETAs) and BAL fluid collected from patients in the intensive care unit (ICU) setting requiring mechanical ventilation (Figure 5A). Variable amounts of chitin were detected in all ETA samples and in a subset of BAL samples, using a chitin blot assay (Figures 5C–5F).^8,25^ In BAL from patients with or at risk for the acute respiratory distress syndrome (Table S1), the presence of chitin was associated with alveolar injury, as alveolar hemorrhage was observed only in samples containing detectable chitin (Figure 5B). Stratifying BAL samples by chitinase activity revealed that samples with the highest chitinase activity contained the lowest chitin amounts (Figure 5C), suggesting that robust chitinase activity degrades environmental chitin in the airways of humans with lung injury. Consistent with this, BAL samples in the high-chitin group also contained increased protein concentrations, indicative of enhanced alveolar permeability and lung injury (Figure 5D). Indeed, chitin particles recovered from BAL and ETAs were susceptible to degradation by a highly active chitinase (CHS; from *T. viride*) compared to control (Figures 5E and 5G). ETA-derived chitin was also differentially degraded by recombinant AMCase isozymes, with the WT variant showing increased particle-degrading activity compared to the hChia enzyme variant (Figures 5H and 5I), in agreement with the differential activity of AMCase isoforms observed on environmental chitin recovered from mouse BAL (Figure S3B).

We next examined *CHIA* genotypes in these patients to determine the contribution of genetic variation to airway chitinase activity in humans. Consistent with the high prevalence of low-activity *CHIA* haplotypes previously reported in diverse human populations,^12^ the majority of individuals (27 out of 35) were homozygous for the trio of hChia-encoding *CHIA* SNPs (G290A, A296G, T339G), whereas 8 individuals were heterozygous for the hChia-encoding *CHIA* SNPs and the mouse-like, DNM-encoding *CHIA* SNPs (A290G, G296A, G339T) (Figure 5J; Table S2). Because both *CHIA* and *CHIT1* (chitotriosidase) can contribute to chitinase activity in humans,^12,26,27^ we additionally evaluated genetic variation in *CHIT1*. We performed genotyping on the same cohort of patient samples to test for the presence of a common 24-bp duplication in *CHIT1*, which has previously been described to impact expression.^28^ In agreement with frequencies reported in prior studies,^28^ 11 of 35 samples were heterozygous for the *CHIT1* duplication, 1 was homozygous for the duplication, and the duplication was absent in the remaining 23 samples (Table S2).

These data suggested that both *CHIA* gene variants and the *CHIT1* gene duplication may contribute to chitinase activity in human tissues. To examine this possibility, we generated a composite chitinase genotype score incorporating the zygosity of both *CHIA* and *CHIT1* variants and related this score to chitinase activity in paired BAL and plasma samples from the same individuals. Chitinase activity in both BAL and plasma significantly correlated with the genotype of both hChia-associated *CHIA* SNPs and the *CHIT1* duplication (Figures 5K and 5L), linking chitinase gene variation with chitinase function in patients with acute lung injury. BAL fluid and plasma chitinase activities were positively correlated across individuals (Figure 5M), indicating that the genetic variance was conserved in chitinase activities in both airway and circulating compartments. Additionally, because we observed an association between reduced AMCase activity and enhanced inflammation in hChia mice, we examined the relationship between AMCase protein amounts and inflammatory cytokines in human BAL samples. In agreement with the results from mouse models, AMCase abundance inversely correlated with IL-1β levels in BAL fluid from patients with acute lung injury (Figure 5N), further supporting the relationship between reduced AMCase function and exacerbated inflammatory cytokine production in the lungs.

Since chitin induces immune triggering via mechanical stretch in the gastrointestinal tract^16^ and prior studies have implicated myeloid cell mechanosensation in the initiation of IL-1β-associated lung inflammation and fibrosis,^29^ we asked whether human airway-derived chitin particles exert similar mechanosensitive inflammatory activity on lung-resident immune cells. We exposed naive WT mouse AMs to intact or chitinase-treated particles recovered from human ETAs and measured expression of inflammatory markers associated with mechanosensation and fibrosis (Figure 5A).^29^ Expression of *Il1b*, *Edn1*, and *Ptgs2* was reduced in AMs exposed to chitinase-treated versus control-treated chitin particles (Figure 5O), indicating that environmental chitin recovered from human airways possesses mechanosensitive inflammatory activity that is attenuated by chitinase treatment. Thus, environmental chitin may represent both a biomarker and a driver of lung disease in humans, which is also susceptible to chitinase-mediated degradation that can mitigate inflammatory exacerbation.

## DISCUSSION

Environmental particles have been implicated in the severity and persistence of a wide range of acute and chronic lung inflammatory conditions marked by clinical heterogeneity.^1–4,30^ Although environmental chitin and chitinases are widespread,^6,9,31^ the influence of these ubiquitous interactions on the mammalian immune system has remained unclear due in part to the recalcitrant nature of chitin particles and the lack of *in vivo* genetic tools to study chitinases. Here, we use translational approaches to show that common genetically encoded human AMCase variants differentially degrade chitin substrates within naturally occurring environmental particles, defining a key gene-environment interaction that underlies lung inflammatory and fibrotic disease heterogeneity. This interaction influences lung inflammatory conditioning in the steady state, lowering the threshold for environmental triggering of resident macrophage and lymphoid cells and subsequently determining susceptibility to IL-1β-associated exacerbations in mouse models of respiratory virus infection, allergic lung disease, and pulmonary fibrosis. Analyses of these pathways in human airway samples revealed that chitin was linked to alveolar hemorrhage and excess airway protein accumulation, suggesting that environmental chitin particles may contribute to lung barrier disruption in settings of human lung injury. The relationship between airway chitinase activity and chitinase gene variation in these individuals further supports a model in which chitinases modulate the influence of environmental chitin on airway inflammatory triggering and susceptibility to lung injury.

Chitin acutely activates lung-resident lymphocytes to recruit mixed inflammatory infiltrates consisting of eosinophils and neutrophils,^20^ hallmarks of severe asthma and COPD (chronic obstructive pulmonary disease) exacerbations,^32–36^ and resembling responses to other crystalline materials such as silica, asbestos,^37,38^ and Gal10 and Ym1 protein crystals, which act as immune adjuvants in the respiratory tract.^39,40^ Our data demonstrating that humanizing hChia mice exhibited prominent AM priming and γδ T cell activation, accompanied by Th17 cell induction after immune challenge, suggest that AMCase enzyme isoforms differentially condition lung-resident immune cells and establish immune adjuvanticity through altered chitin particle degradation. This is further revealed by the increased presence of IFNγ^+^ γδ T cells, T-bet^+^ γδ T cells, and lung DC1 populations in hChia mice, suggesting that altered environmental particle processing promotes a broad inflammatory circuit involving antigen-presenting cells, Th1-associated cytokines, and expansion of tissue-resident innate-like lymphocytes. Similar IFNγ--producing γδ T cell populations accumulate in the fibrotic lungs of aged AMCase-deficient mice and are acutely triggered by IL-1β and IL-23 signaling, further linking impaired chitin degradation to lung inflammatory and fibrotic conditioning.^8,20,41^ Interestingly, despite reduced steady-state ILC2 numbers in hChia lungs, cytokine-driven eosinophil and ILC2 responses to TSLP and IL-33 remained intact, suggesting that environmental particle conditioning alters basal immune tone and lung immune cell composition without intrinsically impairing type 2 effector function in ILC2s, which can be suppressed by heightened interferon signaling in other settings.^42–44^

We also noted persistent IL-1β-associated injury in hChia mice, a pathway implicated in the pathogenesis of fibrosis,^22,24^ particle-driven immune adjuvanticity, and the triggering of mechanosensitive inflammasome pathways in lung innate immune cells.^29^ Notably, increased IL-1β production was evident in the lungs of hChia mice early after bleomycin injury, prior to fibrotic exacerbation and mortality, supporting a role for IL-1β-enhanced inflammatory triggering in driving disease progression in hChia mice. These data are consistent with recent studies demonstrating that IL-1β-driven inflammatory fibroblasts precede pathogenic fibrotic phenotypes during the development of pulmonary fibrosis.^22,24,45,46^ These findings also suggest that incomplete degradation of chitin leaves a persistent environmental imprint on tissue-resident innate cells that then amplifies and exacerbates responses to subsequent insults. Environmental chitin and AMCase activity variants thus determine the extent of inflammatory and fibrotic injury *in vivo*, likely through physical disruption and mechanical stretch as we recently described in other tissues.^16^

Environmental particles contain numerous immunostimulatory components, including β-glucans that are covalently linked to chitin in fungal cell walls.^25,47–49^ Similarly, chitin-containing environmental particles such as those derived from HDMs contain highly crosslinked polysaccharides, chitin-binding proteins, and additional bioactive constituents that may influence substrate accessibility and enzymatic degradation. These complex structural features may contribute to the persistence of environmental chitin *in vivo* despite the presence of endogenous chitinases. This raises the possibility that AMCase isoforms not only regulate chitin clearance but also liberate diverse particle constituents as chitin linkages are cleaved, thereby controlling the tissue dwell time of multiple immunostimulatory and profibrotic elements.^17^

The consequences of impaired chitin degradation may extend beyond acute responses to particle exposure and instead reflect a chronic immune conditioning process that gradually reshapes the responsiveness of lung-resident immune populations. Thus, the exaggerated responses to HDM challenge, viral infection, and bleomycin injury in hChia mice likely arise from altered inflammatory set points established prior to challenge. Although our findings support impaired chitin degradation as the primary mechanism underlying the hChia phenotype, we cannot exclude the possibility that low-activity AMCase variants retain non-chitinolytic functions, including chitin-binding or chi-lectin-like activities, that contribute to immune conditioning or digestive functions that might explain the relatively high prevalence of these AMCase isoforms in humans.^16^ Such mechanisms may account for phenotypic differences between hChia mice and complete AMCase-deficient models^8,10^ and warrant future studies to determine whether therapeutic augmentation of airway chitinase activity can durably reverse established immune conditioning states or prevent their development. Pathways engaged by environmental chitin may thereby impact the turnover and clearance of a disparate array of inorganic and organic materials, including bacteria and viruses,^9,10^ suggesting that lung immunity is broadly shaped in a chitinase-dependent manner. In this context, the diminishment of inflammatory cell responses following enzymatic degradation of environmental chitin particles recovered from humans with lung injury suggests that chitinase-based therapy may both limit lung disease severity and reduce diverse environmentally driven exacerbations.

### Limitations of the study

This study has several limitations. First, because environmental chitin exposure varies among individual animals housed under standard conditions, airway chitin burden provides only a snapshot of a dynamic process and may underestimate cumulative differences in chitin turnover over time. Although we primarily quantify chitin, additional immunostimulatory elements (e.g., endotoxins, antigenic proteins, and glucans) may be complexed with chitin in naturally occurring environmental particles, and their turnover in lung tissues may also depend on chitinase activity, thus potentially contributing to the immune conditioning that we describe. Second, although our genetically engineered mouse models demonstrate that reduced AMCase activity promotes environmentally driven lung immune conditioning, the human analyses are primarily correlative and therefore cannot establish the same relationships *in vivo*. Finally, the human cohort was relatively small and comprised critically ill patients with acute respiratory failure, limiting statistical power and the generalizability of these findings. Larger prospective studies across broader patient populations will be important to further define the relationships among *CHIA* genotype, chitinase activity, environmental chitin burden, and respiratory disease.

## RESOURCE AVAILABILITY

### Lead contact

Requests for further information and resources should be directed to and will be fulfilled by the lead contact, Steven J. Van Dyken (svandyken@wustl.edu).

### Materials availability

The humanized hChia mouse lines generated in this study are available via materials transfer agreement with Washington University and the lead contact upon reasonable request.

### Data and code availability

- All data reported in this paper will be shared by the lead contact upon request.
- This paper does not report original code.
- Any additional information required to reanalyze the data reported in this paper is available from the lead contact upon request.

## STAR★METHODS

### EXPERIMENTAL MODEL AND STUDY PARTICIPANT DETAILS

#### Mice

Humanizing Chia (hChia) mice were generated by CRISPR/Cas9-mediated gene editing in mouse C57BL/6J mouse zygotes, using a single gRNA and donor ssODN to incorporate two-point (G290A/A296G) and three-point (G290A/A296G/T339G) mutations (Figure 1A). Correctly targeted point mutants were verified by sequencing and heterozygous founders were crossed with wild-type (WT) C57BL/6J mice to generate homozygous hChia and WT controls, which were maintained under specific pathogen-free (SPF) conditions. Male mice were used between 8 and 12 weeks of age and under standard conditions using autoclaved corn cob bedding (Andersons), unless otherwise noted. Where indicated, mice were housed using low-chitin bedding (ALPHA-dri PLUS; Shepherd Specialty Papers), composed of low-dust-emitting virgin paper pulp cellulose squares. All procedures were approved by the Washington University School of Medicine in St. Louis Institutional Animal Care and Use Committee (Protocol #24–0051, Animal Welfare Assurance #D16–00245).

#### Human study participants

Endotracheal aspirates (ETA) were collected from mechanically ventilated intensive care unit (ICU) patients as part of routine care. As the ETA samples were set aside without any way for the study team to link them to individuals or identifiable information, the study was determined to not meet the definition of human subjects by the Washington University Human Research Protections Office (HRPO# 202407022). In separate human subjects studies, leftover bronchoalveolar lavage (BAL) samples were obtained from an ongoing bio-repository of critically ill patients who underwent medically indicated fiberoptic bronchoscopy with acute respiratory failure and pulmonary infiltrate(s) with or at risk for the acute respiratory distress syndrome (IRB#202310214). Participant demographic and clinical characteristics are summarized in Table S1. An IRB approval was obtained to utilize de-identified BAL samples from the bio-repository for the purpose of examining chitin and chitinases in acute lung injury and limited clinical information (IRB# 202507042). Peripheral blood samples were obtained from a subset of participants enrolled in the BAL bio-repository study described above. Bronchoalveolar lavage samples were obtained from 39 participants.

CHIA and CHIT1 genotyping was performed in 35 individuals with sufficient DNA available, and PBMCs were obtained from a subset of participants.

### METHOD DETAILS

#### *In vivo* treatments

House dust mite (HDM) crude preparation (from *Dermatophagoides pteronyssinus*; HollisterStier Allergy) was passed through a 75-μm filter and resuspended in Ca^2+^/Mg^2+^-free phosphate-buffered saline (PBS; Leinco). Mice were lightly anesthetized with isoflurane and 50 μL of HDM (1 mg/mL in PBS) was intranasally administered every 3 days, for a total of four instillations, followed by euthanasia 24 h after the final dose for tissue harvest. Inoculation of influenza A virus [IAV; A/Puerto Rico/8/1934 (H1N1)], kindly provided by A. Boon (Washington University School of Medicine), was performed as described.^10^ Briefly, mice were anesthetized with isoflurane, intranasally inoculated with 30 μL IAV (250 plaque-forming units) or PBS, weighed every two days, and in some cases received HDM (50 μL, 0.2 mg/mL) at 34 and 36 days post-IAV inoculation (dpi), as indicated. Bleomycin (Cayman Chemical) was reconstituted in PBS and intranasally administered to anesthetized mice at a dose of 2 mg/kg. Animals were weighed every three days and euthanized if weight loss exceeded 30% of initial body weight. In some experiments, mice were intranasally instilled with bleomycin as described above, then subsequently challenged with HDM (50 μL; 1 mg/mL in PBS) every 2 days, followed by euthanasia and tissue collection at day 23 post-bleomycin administration. For acute cytokine stimulation, mice were anesthetized with isoflurane and intranasally administered recombinant IL-1β (R&D Systems) and IL-23 (BioLegend), 10 ng each cytokine in PBS, followed by euthanasia 18 h after cytokine administration for tissue collection and analysis.

#### Tissue preparation and flow cytometry

Mouse serum was separated from venous blood by allowing blood to clot at room temperature for one hour before centrifuging at 7000 g for 10 min and collecting supernatant. Bronchoalveolar lavage (BAL) was performed by intratracheal instillation of PBS (1 mL), which was recovered and centrifuged at 3000 rpm for 10 min, then separated into pellet and supernatant fractions for further analysis. Stomachs were opened longitudinally, then vortexed in 1mL PBS to separate tissue from contents, which were centrifuged at 10000 g for 10 min. Supernatants comprising stomach lavage were collected and tissue was further prepared for flow cytometry analysis. For flow cytometry, spleens and lymph nodes were mechanically dissociated in 2% FBS in PBS, passed over 70-μm mesh followed by red blood cell lysis (Fisher) to generate cell suspensions prior to antibody staining. For lung preparation, mice were perfused via cardiac puncture with 10 mL PBS before lungs were dissected and enzymatically digested in Hanks’ balanced salt solution (HBSS; Fisher) containing Liberase TM (25 μg/mL; Roche) and DNase I (5 μg/mL; Roche) using C tubes (Miltenyi Biotec). Tissues were processed using an automated tissue dissociator (gentleMACS; Miltenyi Biotec) to generate single-cell suspensions, which were subsequently stained with antibodies listed in the key resources table. Flow cytometric gating strategies are shown in Figure S2. For detection of HDM-specific T cells, class II MHC tetramers reactive to house dust mite Der p 1 (IEDB ID: 242387) or control human CLIP (IEDB ID: 119507; NIH Tetramer Core Facility) were used to stain single-cell suspensions following tissue dissociation. Gastric and intestinal tissues were processed as previously described.^16^ Briefly, tissues were incubated in HBSS supplemented with Ca^2+^/Mg^2+^ (Fisher), 10 mM HEPES (Fisher), 5 mM DTT (GoldBio), and 2% fetal bovine serum (FBS, Gibco) at 37°C to remove epithelial cells, followed by two sequential incubations in HBSS containing 10 mM HEPES, 5 mM EDTA (Fisher), and 2% FBS. EDTA fractions were discarded. Remaining tissue fragments were washed, minced, and enzymatically digested in HBSS containing 10 mM HEPES, 2% FBS, Liberase TM (25 μg/mL), and DNase I (5 μg/mL). Single-cell suspensions were filtered through a 100-μm mesh, washed, and stained for flow cytometry. For small intestinal samples, immune cells were further enriched by centrifugation on a 40–70% Percoll gradient (Sigma-Aldrich). For intracellular cytokine staining, lung single-cell suspensions were enriched for lymphocytes using a Percoll gradient. Cells were stimulated in RPMI 1640 supplemented with 10% FBS, 50 ng/mL PMA (Sigma), and 500 ng/mL ionomycin (Sigma) in the presence of brefeldin A (BioLegend) for 4 h at 37°C. Following stimulation, cells were washed with PBS and stained for viability using Live/Dead Blue dye (1:1000) for 15 min at room temperature, followed by surface marker staining in FACS buffer for 30 min on ice. Cells were subsequently fixed and permeabilized using the Foxp3/Transcription Factor Staining Buffer Set (eBioscience) and stained overnight at 4°C with antibodies against cytokines and transcription factors prior to flow cytometric analysis. Data were acquired on a BD FACSymphony A3 flow cytometer and analyzed with FlowJo software (BD Biosciences).

#### Histology and immunofluorescence

Left lung lobes were removed and fixed in 4% paraformaldehyde (PFA, Santa Cruz Biotechnology), washed in PBS, then incubated in 30% sucrose (sterile-filtered after dissolving in PBS) at 4°C for 48 h. Tissue was embedded in optimal cutting temperature compound (OCT, Sakura) prior to cryosectioning. For immunofluorescence, cryosections were blocked in PBS containing 2% bovine serum albumin (BSA, Sigma-Aldrich), Fc block (1:1000, Bio X Cell), and normal goat serum (Thermo Fisher Scientific), for 2 h at room temperature. Sections were incubated at 4°C overnight with primary antibodies in blocking buffer: conjugated anti-αSMA (1:1000; Cell Signaling Technology), or anti-CTHRC1 (1:250; MaineHealth Research Institute). Sections were then washed in PBS (3 × 5 min) and incubated with secondary antibody (anti-rabbit Alexa Fluor 555, 1:2000 in blocking buffer; Thermofisher) for 1 h at room temperature. After three additional washes with PBS, slides were air-dried for 20 min and rehydrated in PBS for 5–10 min. Nuclear counterstaining was performed with DAPI (1:1000 in blocking buffer), then slides were rinsed with PBS and mounted with Fluoromount (Thermo Fisher Scientific).

#### Human endotracheal aspirate (ETA), BAL, and PBMC collection

ETA samples were collected as part of routine suctioning by instilling 5 mL of 0.9% sodium chloride (normal saline) through the endotracheal tube followed by advancing the in-line suction catheter and retrieving airway secretions into a closed specimen collection system. ETA samples were stored on ice until further processing. For the chitin and chitinase assays, ETA samples were centrifuged at 20,000 g × 20 min to separate the supernatant and pellet fractions for downstream analysis. Leftover BAL fluid was obtained following standard clinical bronchoscopy procedures that utilized serial instillation of normal saline into a subsegmental bronchus selected by and performed by the clinical provider team. After collection, BAL samples were kept on ice and were gently pipetted over a sterile 70- μm nylon mesh filter to remove cellular debris, centrifuged at 300 g × 5 min at 4°C, and the supernatant was collected and cryopreserved at – 80°C until further use. BAL samples were subsequently centrifuged at 20,000 *g* × 20 min to separate the supernatant and pellet fractions for downstream analysis. Whole blood was collected in EDTA tubes and centrifuged at 1,000 g × 10 min to separate plasma from the cellular fraction. The remaining blood cells were resuspended in 30 mL sterile phosphate-buffered saline (PBS) and layered over a Ficoll density gradient using SepMate tubes for peripheral blood mononuclear cell (PBMC) isolation. Samples were centrifuged at 1,200 g × 20 min at room temperature, and the PBMC layer was carefully collected, washed with sterile PBS, counted using an automated cell counter, and pelleted by centrifugation at 300*g* × 10 min. PBMCs were cryopreserved in CryoStor CS10 at a concentration of 1 × 10^7^ cells/mL. Cryopreservation was initiated using a controlled-rate freezing container at −80°C prior to transfer to liquid nitrogen for long-term storage.

#### *In vitro* macrophage stimulation

Alveolar macrophages (AMs) were prepared as described.^51^ After isolation, cells were resuspended in complete RPMI 1640 supplemented with GlutaMAX, penicillin–streptomycin (Fisher), sodium pyruvate (Corning), and 10% fetal bovine serum (FBS). Cells were counted by trypan blue exclusion, seeded at approximately 5×10^4^ cells per well in 12-well plates, allowed to adhere overnight at 37°C in 5% CO_2_. For particle stimulation experiments, human endotracheal aspirate (ETA) pellets were heat-treated at 95°C for 10 min to inactivate and denature proteins, then incubated with chitinase from Trichoderma viride (Sigma) at 1 mg/mL. Particulate suspensions were mixed 1:1 (v/v) with chitinase or PBS control and incubated at 37°C for 72 h, then were heat-inactivated at 95°C for 10 min. Following incubation, 50 μL of control or chitinase-treated ETA particles were added to each well of cultured alveolar macrophages, incubated for 6 h at 37°C in 5% CO_2_ and cell pellets were harvested for RNA isolation and quantitative PCR analysis.

#### RNA isolation and qPCR

Total RNA was isolated from cultured alveolar macrophages using the RNeasy Plus Mini Kit (Qiagen) and converted to cDNA using the SuperScript IV VILO Master Mix (Thermo Fisher Scientific) according to the manufacturer’s instructions. Quantitative PCR (qPCR) was performed using Power SYBR Green PCR Master Mix (Thermo Fisher Scientific) on a CFX Connect Real-Time PCR System (Bio-Rad). Gene expression levels were normalized to the ribosomal gene *Rps17*, and relative transcript abundance was calculated using the comparative ΔΔCt method (2^∧− ΔΔCt^). Primer sequences (5′–3′) were as follows:

*Edn1*: (F)GCACCGGAGCTGAGAATGG; (R)GTGGCAGAAGTAGACACACTC.

*Il1b*: (F)GCAACTGTTCCTGAACTCAACT; (R)ATCTTTTGGGGTCCGTCAACT.

*Ptgs2*: (F)GCGACATACTCAAGCAGGAGCA; (R)AGTGGTAACCGCTCAGGTGTTG.

*Rps17*: (F)CGCCATTATCCCCAGCAAG; (R)TGTCGGGATCCACCTCAATG.

*Chia1*: (F) TACCAGACAGGCTGGGTTCT; (R) GGAGTAGTCACTGGCTCGGA.

*Il13*: (F)CCTGGCTCTTGCTTGCCTT; (R) GGTCTTGTGTGATGTTGCTCA.

*Il17a*: (F)TTTAACTCCCTTGGCGCAAAA; (R) CTTTCCCTCCGCATTGACAC.

#### Biochemical assays

Recombinant WT and hChia AMCase variants were prepared in the laboratory of J. Fraser (UCSF) as described^14,50^ and chitinase activity assays were performed as in prior studies.^8,16^ Briefly, for activity measurements on soluble substrates, 4-methylumbelliferyl-N,N′-diacetylchitobioside hydrate (Sigma) was diluted to 20 μg/mL final concentration in McIlvaine buffer (pH 5.0) and incubated with 5μL of biological fluid samples at 37°C for 30 min. The reaction was stopped by sodium carbonate solution (40 μg/mL), and fluorescence was measured on a Synergy H1 microplate reader (Agilent). Chitinase activity on insoluble substrates was measured using a modified ChitO-HRP coupled assay, based on previously described methods.^14–16^ Insoluble colloidal chitin particles (Megazyme) were washed 10 times with an excess of McIlvaine buffer (pH 7.0) to remove residual soluble N-acetylglucosamine (GlcNAc) and free GlcNAc oligosaccharides. Washed chitin particles were resuspended at 1 mg/mL in McIlvaine buffer and incubated with biological fluids (1:1 volume ratio) for 3 h at 37°C. The reaction was terminated by heating the mixture for 10 min at 95°C. To quantify GlcNAc released from chitin degradation, 50 μL of the reaction mixture was combined with 50 μL of detection solution containing 10 nM chitinase (if recombinant protein was used), 20 U/mL horseradish peroxidase (HRP), 100 nM chito-oligosaccharide oxidase (ChitO), 0.5 μL of ADHP substrate, and 10 μL of QuantaRed enhancer solution, all in McIlvaine buffer (pH 7.0). Fluorescence was measured using a Synergy H1 microplate reader (Agilent) to determine GlcNAc release as a readout of chitinase activity. To monitor chitin particle degradation, 0.25% insoluble colloidal chitin particles (Megazyme) were incubated with bronchoalveolar lavage (BAL) fluid (1:1 volume ratio). Chitin-BAL mixtures were incubated at 37°C for 72 h. Following incubation, residual chitin particles were imaged using an EVOS M7000 Imaging System (Thermo Fisher Scientific). Chitin blotting was performed as previously described.^8^ Chitin-containing samples were spotted onto nitrocellulose membranes, air-dried overnight, and blocked in 5% BSA in TBST for 1 h at room temperature. Membranes were incubated overnight at 4°C with CBD-eGFP (1:2000 in blocking buffer), washed, and probed with biotin-conjugated anti-GFP (1:2000; Invitrogen) for 1 h at room temperature. After additional washes, membranes were incubated with streptavidin-HRP (1:4000; Sigma-Aldrich) for 45 min, washed, and chemiluminescent signal was generated using ECL substrate (Bio-Rad). Band intensities were quantified by densitometry and expressed as relative units (RU). Mouse IL-1β, IL-5, IL-6, IL-13, IL-17A, TNF-α, HDM-specific IgE, and human IL-1β were measured using commercially available ELISA kits according to the manufacturers’ instructions (see key resources table).

### QUANTIFICATION AND STATISTICAL ANALYSIS

Data are presented as mean ± SEM, and results from independent experiments were pooled unless otherwise indicated. Individual data points represent biological replicates. Exact sample sizes (n) and statistical tests used for each experiment are indicated in the corresponding figure legends; for animal experiments, n represents individual mice, and for human studies, n represents individual participant samples. Statistical analyses were performed using Prism (GraphPad Software). *p* values were calculated using unpaired two-tailed *t* test, paired *t* test, one-way or two-way analysis of variance (ANOVA) followed by appropriate post hoc tests, mixed-effects models with restricted maximum likelihood (REML) for repeated-measures data where appropriate, Fisher’s exact test, or Spearman correlation analysis, as specified in the figure legends. Statistical significance was defined as *p* < 0.05. Animals were sex-matched between experimental groups.

## Supplementary Material

1

SUPPLEMENTAL INFORMATION

Supplemental information can be found online at https://doi.org/10.1016/j.celrep.2026.117833.

## Figures and Tables

**KEY RESOURCES TABLE:** 

REAGENT or RESOURCE	SOURCE	IDENTIFIER
Antibodies		
*DAPI*	*BioLegend*	Cat#422801
*Anti-mouse CD45 antibody (clone 30-F11)*	*BD Biosciences*	Cat#564279; RRID: AB_2651134
*Anti-mouse CD3 antibody (clone 17A2)*	*BioLegend*	Cat#100220; RRID:AB_1732057
*Anti-mouse CD3 antibody (clone 145–2C11)*	*BioLegend*	Cat#100341; RRID: AB_2562556
*Anti-mouse CD4 antibody (clone RM4–5)*	*BioLegend*	Cat#100557; RRID: AB_2562607
*Anti-mouse CD4 antibody (clone RM4–5)*	*BioLegend*	Cat#100532; RRID: AB_493373
*Anti-mouse CD8α antibody (clone 53–6.7)*	*BD Biosciences*	Cat#612898; RRID: AB_2870186
*Anti-mouse TCRβ antibody (clone H57–597)*	*BioLegend*	Cat#109220; RRID: AB_893624
*Anti-mouse TCRγδ antibody (clone GL3)*	*BioLegend*	Cat#118118; RRID: AB_10612756
*Anti-mouse TCRγδ antibody (clone GL3)*	*BioLegend*	Cat#118136; RRID: AB_2650828
*Anti-mouse TCRγδ antibody (clone GL3)*	*BD Biosciences*	Cat#755465; RRID: AB_3687834
*Anti-mouse CD19 antibody (clone 6D5)*	*BioLegend*	Cat#115578
*Anti-mouse NK1.1 antibody (clone PK136)*	*BD Biosciences*	Cat#758960; RRID: AB_3691063
*Anti-mouse CD90.2 antibody (clone 53–2.1)*	*BD Biosciences*	Cat#741701; RRID: AB_2813887
*Anti-mouse CD90.2 antibody (clone 53–2.1)*	*BioLegend*	Cat#140318; RRID: AB_2650924
*Anti-mouse CD25 antibody (clone PC61)*	*BioLegend*	Cat#102007; RRID: AB_312856
*Anti-mouse CD44 antibody (clone IM7)*	*BioLegend*	Cat#103044; RRID: AB_2650923
*Anti-mouse CD69 antibody (clone H1.2F3)*	*BioLegend*	Cat#104516; RRID: AB_492845
*Anti-mouse CD69 antibody (clone H1.2F3)*	*BioLegend*	Cat#104543; RRID: AB_2629640
*Anti-mouse KLRG1 antibody (clone 2F1/KLRG1)*	*BioLegend*	Cat#138429; RRID: AB_2629749
*Anti-mouse ST2 antibody (clone RMST2–2)*	*Invitrogen, eBioscience*	Cat#46–9335–80
*Anti-mouse CD11b antibody (clone M1/70)*	*BioLegend*	Cat#101259; RRID: AB_2566568
*Anti-mouse CD11b antibody (clone M1/70)*	*BioLegend*	Cat#101257; RRID: AB_2565431
*Anti-mouse CD11c antibody (clone N418)*	*BioLegend*	Cat#117336; RRID: AB_2565268
*Anti-mouse CD11c antibody (clone N418)*	*BioLegend*	Cat#117339; RRID: AB_2562414
*Anti-mouse CD11c antibody (clone N418)*	*BD Biosciences*	Cat#749040; RRID: AB_2873434
*Anti-mouse Ly6G antibody (clone 1A8)*	*BioLegend*	Cat#127624; RRID: AB_10640819
*Anti-mouse I-A/I-E (MHC II) antibody (clone M5/114.15.2)*	*BioLegend*	Cat#107643; RRID: AB_2565976
*Anti-mouse Siglec-F antibody (clone E50–2440)*	*BD Biosciences*	Cat#562680; RRID: AB_2687570
*Anti-mouse Siglec-F antibody (clone E50–2440)*	*BD Biosciences*	Cat#565526; RRID: AB_2739281
*Anti-mouse EpCAM (CD326) antibody (clone G8.8)*	*BioLegend*	Cat#118215; RRID: AB_1236477
*Anti-mouse IL-4 antibody (clone 11B11)*	*BioLegend*	Cat#504133; RRID: AB_2565950
*Anti-mouse IL-17A antibody (clone TC11–18H10.1)*	*BioLegend*	Cat#506930; RRID: AB_2686975
*Anti-mouse IL-13 antibody (clone eBio13A)*	*Invitrogen, eBioscience*	Cat#12–7133–81
*Anti-mouse IL-5 antibody (clone TRFK5)*	*BioLegend*	Cat#504305; RRID: AB_315329
*Anti-mouse IFN-γ antibody (clone XMG1.2)*	*BioLegend*	Cat#505838; RRID: AB_2629667
*Anti-mouse RORγt antibody (clone Q31–378)*	*BD Biosciences*	Cat#562683; RRID: AB_2737720
*Anti-mouse RORγt antibody (clone AFKJS-9)*	*Invitrogen, eBioscience*	Cat#61–6988–80
*Anti-mouse T-bet antibody (clone 4B10)*	*BioLegend*	Cat#644841; RRID: AB_3662326
*Anti-mouse GATA3 antibody (clone 16E10A23)*	*BioLegend*	Cat#653819; RRID: AB_3083421
*Anti-mouse Foxp3 antibody (clone FJK-16s)*	*Invitrogen, eBioscience*	Cat#56–5773–80
*Anti-α-smooth muscle actin (αSMA) antibody (clone D4K9N)*	*Cell Signaling Technology*	Cat#CSIG-34105S
*Anti-CTHRC1 rabbit monoclonal antibody*	*MaineHealth Research Institute*	Cat#VLi55
*Goat anti-rabbit Alexa Fluor 555 secondary antibody*	*Thermofisher*	Cat#A-21428
*Fc Block (anti-mouse CD16/CD32)*	*Bio X Cell*	Cat#BE0307; RRID: AB_2736987
*Biotin-conjugated anti-GFP antibody*	*Invitrogen*	Cat#13–6498–82
*Streptavidin-HRP*	*Sigma-Aldrich*	Cat#RABHRP3–600UL
Bacterial and virus strains		
*Influenza A virus, A/Puerto Rico/8/1934 (H1N1) (PR8)*	*A. Boon (Washington University School of Medicine)*	*N/A*
Biological samples		
*Human endotracheal aspirate (ETA) samples*	*This paper*	*N/A*
*Human bronchoalveolar lavage (BAL) samples*	*This paper*	*N/A*
*Human peripheral blood mononuclear cells (PBMCs)*	*This paper*	*N/A*
Chemicals, peptides, and recombinant proteins		
*House dust mite (HDM) extract (Dermatophagoides pteronyssinus)*	*HollisterStier Allergy*	*N/A*
*Bleomycin sulfate*	*Cayman Chemical*	*Cat#13877*
*Recombinant mouse IL-1β*	*R&D*	*Cat#401-ML-005/CF*
*Recombinant mouse IL-23*	*BioLegend*	*Cat#589002*
*Recombinant wild-type (WT) AMCase*	*Barad*et al.,^14^; *Díaz*et al.,^50^	*N/A*
*Recombinant hChia AMCase*	*Barad*et al.,^14^; *Díaz*et al.,^50^	*N/A*
*Chitinase from Trichoderma viride*	*Sigma-Aldrich*	*Cat#C8241*
*Insoluble colloidal chitin*	*Megazyme*	*Cat#P-CHITN*
*4-Methylumbelliferyl N,N*′*-diacetylchitobioside hydrate*	*Sigma-Aldrich*	*Cat#M9763–5MG*
*Chito-oligosaccharide oxidase (ChitO)*	*Gecco Biotech*	*N/A*
*Horseradish peroxidase (HRP)*	*Thermo Fisher Scientific*	*Cat#15159*
*ADHP substrate*	*Thermo Fisher Scientific*	*Cat#15159*
*Chitin binding domain (CBD)-eGFP*	*J. Fraser (University of California, San Francisco; UCSF)*	*N/A*
*Der p 1 MHC class II tetramer*	*NIH Tetramer Core Facility*	*IEDB ID: 242387*
*Human CLIP control MHC class II tetramer*	*NIH Tetramer Core Facility*	*IEDB ID: 119507*
Critical commercial assays		
*RNeasy Plus Mini Kit*	*Qiagen*	*Cat#74136*
*SuperScript IV VILO Master Mix*	*Thermo Fisher Scientific*	*Cat#11766050*
*Power SYBR Green PCR Master Mix*	*Thermo Fisher Scientific*	*Cat#4367659*
*HDM-specific IgE ELISA kit*	*Chondrex*	*Cat#3037*
*Mouse IL-1β ELISA Kit*	*Biolegend*	*Cat#432604*
*Mouse TNF-α ELISA Kit*	*Biolegend*	*Cat#430904*
*Mouse IL-5 ELISA Kit*	*Biolegend*	*Cat#431204*
*Mouse IL-13 ELISA Kit*	*Thermo Fisher Scientific*	*Cat#88–7137–88*
*Mouse IL-17A ELISA Kit*	*R&D*	*Cat#DY421–05*
*Mouse IL-6 ELISA Kit*	*R&D*	*Cat#DY406–05*
*Human IL-1β ELISA Kit*	*Biolegend*	*Cat#437004*
*eBioscience*^*TM*^ *Foxp3/Transcription Factor Staining Buffer Set*	*Thermo Fisher Scientific*	*Cat#00–5523–00*
Experimental models: Organisms/strains		
*C57BL/6J mice*	*The Jackson Laboratory*	*JAX: 000664*
*Humanized Chia (hChia) mice*	*This paper*	*N/A*
*Humanized Chia two-point (hChia-2pt) mice*	*This paper*	*N/A*
Oligonucleotides		
*Edn1 forward primer*	*Sigma-Aldrich*	*See* STAR Methods
*Edn1 reverse primer*	*Sigma-Aldrich*	*See* STAR Methods
*Il1b forward primer*	*Sigma-Aldrich*	*See* STAR Methods
*Il1b reverse primer*	*Sigma-Aldrich*	*See* STAR Methods
*Ptgs2 forward primer*	*Sigma-Aldrich*	*See* STAR Methods
*Ptgs2 reverse primer*	*Sigma-Aldrich*	*See* STAR Methods
*Rps17 forward primer*	*Sigma-Aldrich*	*See* STAR Methods
*Rps17 reverse primer*	*Sigma-Aldrich*	*See* STAR Methods
*Chia1 forward primer*	*Sigma-Aldrich*	*See* STAR Methods
*Chia1 reverse primer*	*Sigma-Aldrich*	*See* STAR Methods
*Il13 forward primer*	*Sigma-Aldrich*	*See* STAR Methods
*Il13 reverse primer*	*Sigma-Aldrich*	*See* STAR Methods
*Il17a forward primer*	*Sigma-Aldrich*	*See* STAR Methods
*Il17a reverse primer*	*Sigma-Aldrich*	*See* STAR Methods
Software and algorithms		
*FlowJo v10*	*BD Biosciences*	*N/A*
*GraphPad Prism v11*	*GraphPad Software*	*N/A*
*Image Lab*	*Bio-Rad*	*N/A*
*Fiji (ImageJ)*	*National Institutes of Health*	*N/A*
*QuPath*	*QuPath developers*	*N/A*
*ZEN*	*Zeiss*	*N/A*

## Highlights

- Humanized (hChia) mice recapitulate common human AMCase activity variants *in vivo*
- Basal lung inflammatory tone is elevated in low-activity hChia mice
- Allergic, viral, and fibrotic lung disease is exacerbated in low-activity hChia mice
- Chitinase reduces inflammatory effects of chitin from patients with acute lung injury
