# Supplementary material for: Humanizing acidic mammalian chitinase variants establish lung immune conditioning and control environmentally driven inflammation and fibrosis
Source: Cell Rep. Author manuscript; Available in PMC 2026 Sep 23. (PMC13600261; doi:10.1016/j.celrep.2026.117833)
Supplement: Supplementary file 1 [file NIHMS2206924-supplement-1.pdf]

Cell Reports, Volume *45*

Supplemental information

Humanizing acidic mammalian chitinase variants establish lung immune conditioning and control environmentally driven inflammation and fibrosis

Yilin Wang, Haerin Jung, Do-Hyun Kim, Eilene Liu, Donovan Watza, Samuel I. Risma, Kira L. Florczak, Leah M. Kim, Zeena Bou Reslan, Ananya Gupta, Ruppal Soni, Roberto Efraín Díaz, Richard M. Locksley, James S. Fraser, Janet S. Lee, and Steven J. Van Dyken

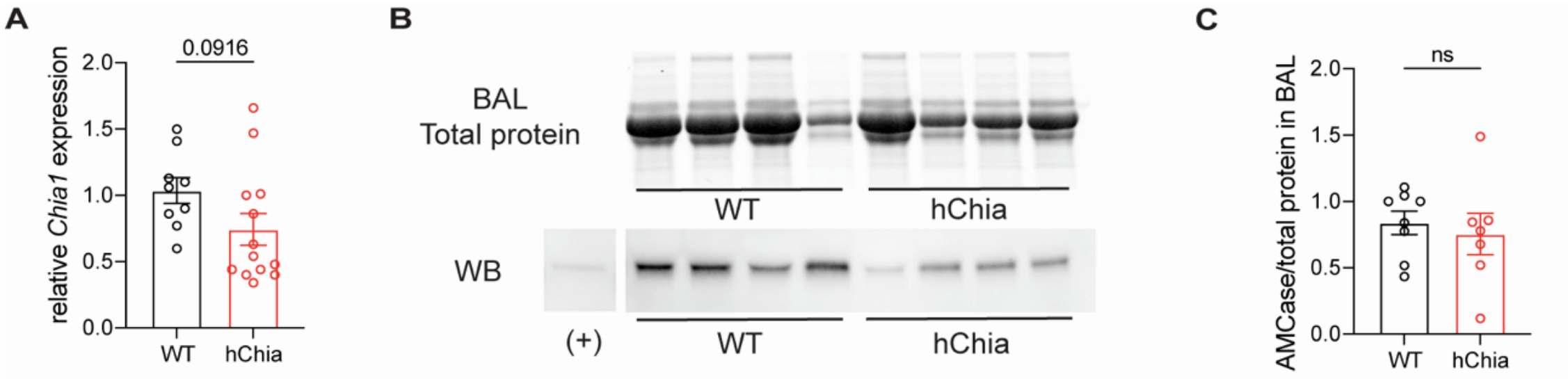

Figure S1. Basal AMCase expression in WT and hChia mice

(A), Relative Chia1 mRNA expression in whole lung homogenates from steady-state WT and hChia mice measured by qPCR. (B), Representative western blot analysis of AMCase protein levels in BAL fluid from WT and hChia mice. Equal BAL fluid volumes were loaded, and total protein staining is shown above the AMCase immunoblot. Positive control recombinant AMCase protein is shown at left. (C), Quantification of AMCase protein normalized to total BAL protein from western blot analysis shown in (B). Data are presented as mean ± SEM and are pooled from at least 2 independent experiments; individual data points represent biological replicates. Statistical analyses were performed using unpaired t-test (A, C). Ns, not significant.

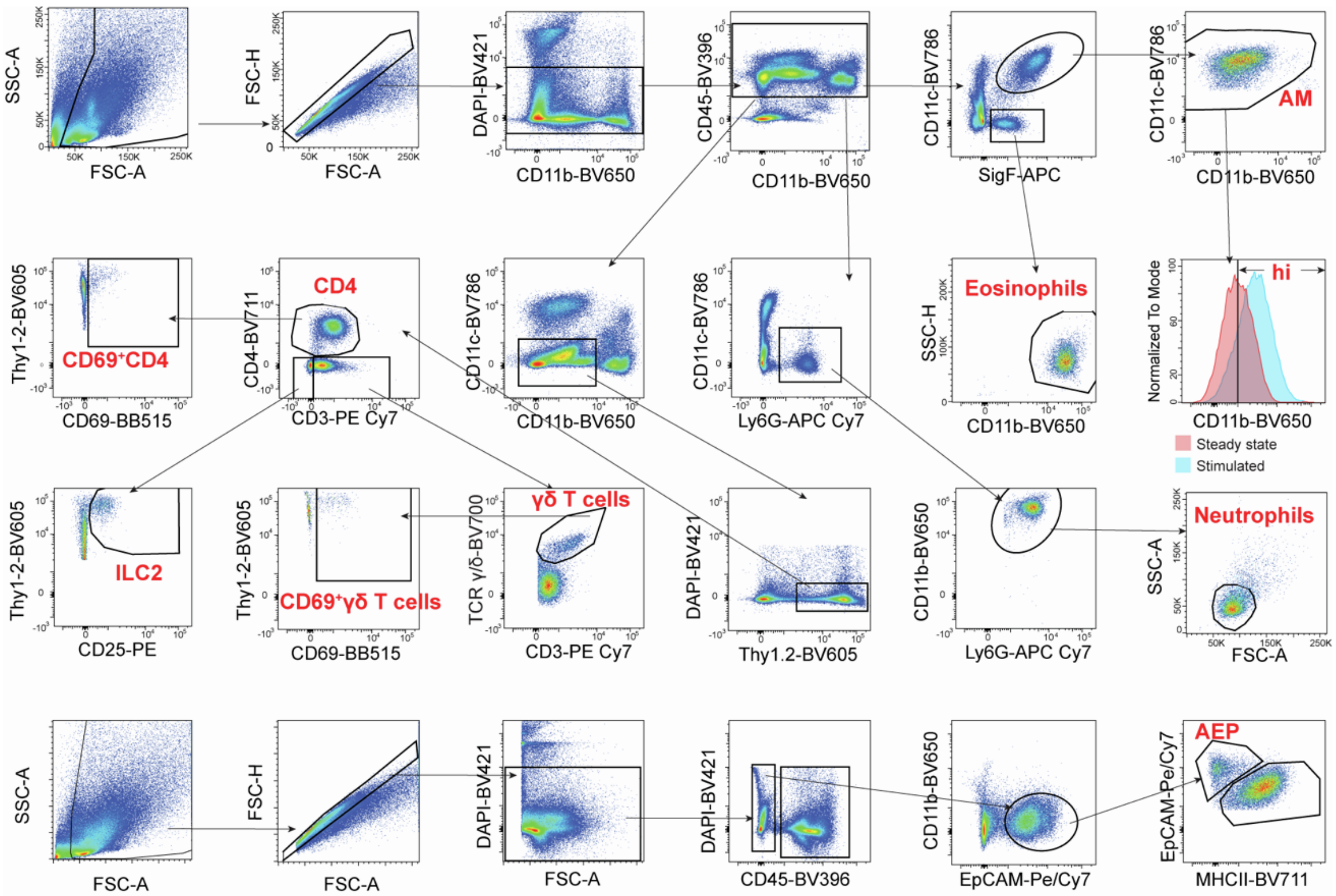

Figure S2. Flow cytometry gating

Single-cell suspensions from mouse lungs were analyzed by flow cytometry to identify indicated immune cell populations. Debris, doublets, and dead cells were excluded based on FSC/SSC, FSC-A vs. FSC-H, and DAPI staining, respectively. Representative panel from steady state and IL-1β + IL-23 stimulation indicates CD11bhi alveolar macrophage (AMs; CD11c^{+}SiglecF^{+}) gating.

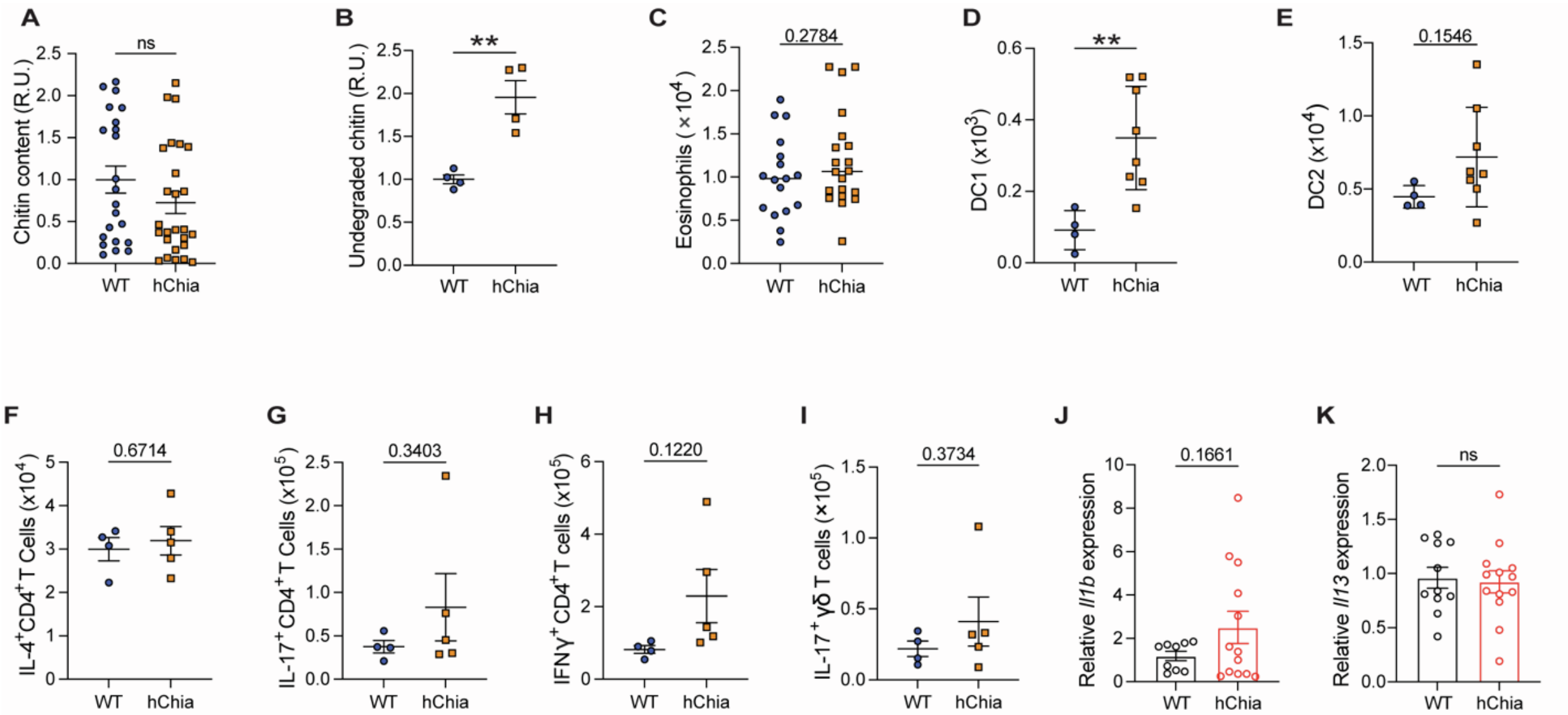

Figure S3. Airway chitin and immune cells in lungs of WT and hChia mice

(A), Bronchoalveolar lavage (BAL) chitin content in wild-type (WT) and hChia mice. (B), BAL chitin remaining after incubation with recombinant WT or hChia variant isozymes. (C-E), Quantification of eosinophils (C), DC1 (D), and DC2 (E) populations in lung tissue from steady-state WT and hChia mice by flow cytometry. (F-I) Quantification of IL-4$^+$ (F), IL-17$^+$ (G), IFNγ$^+$ (H) CD4$^+$ T cells and IL-17$^+$ γδ T cells (I) in lung tissue following cytokine staining and flow cytometric analysis. (J-K) Relative expression of Il1b and Il13 in whole lung homogenates measured by qPCR. Data are presented as mean ± SEM and are pooled from at least 2 independent experiments; individual data points represent biological replicates. Statistical analyses were performed using unpaired t-test. **$p < 0.01$; ns, not statistically significant.

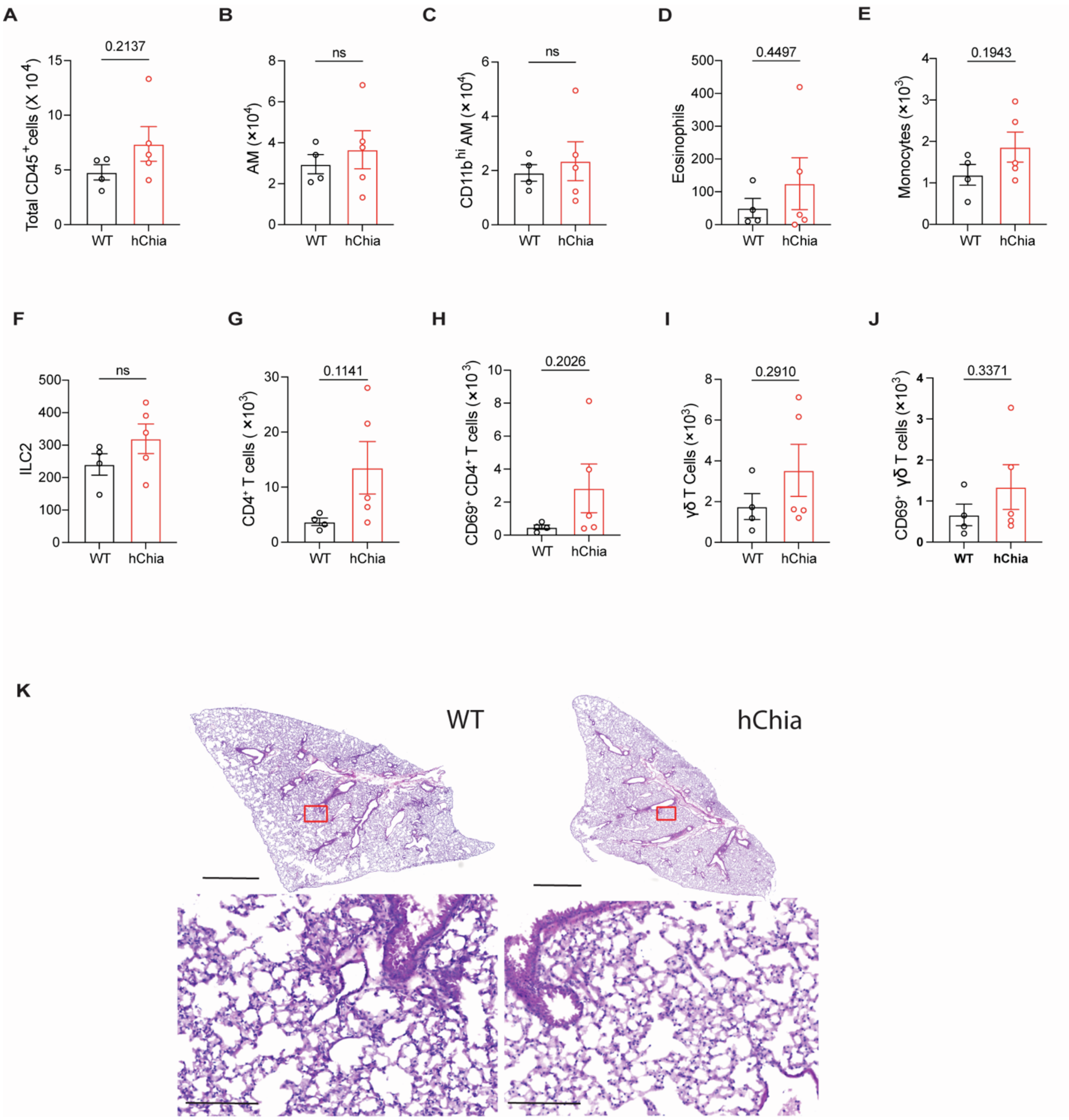

Figure S4. Steady-state BALF immune profiling and lung histology in WT and hChia mice

(A-J), Flow cytometric quantification of total CD45$^+$ cells (A), alveolar macrophages (AM, B), CD11b^{hi} alveolar macrophages (C), eosinophils (D), monocytes (E), ILC2s (F), CD4$^+$ T cells (G), CD69$^+$ CD4$^+$ T cells (H), γδ T cells (I), and CD69$^+$ γδ T cells (J) in bronchoalveolar lavage fluid (BALF) from steady-state WT and hChia mice. (K), Representative H&E-stained lung sections from steady-state WT and hChia mice shown at low magnification (top) and higher magnification (bottom, boxed regions). Scale bars, top: 2 mm; bottom: 200 μm. Data are presented as mean ± SEM, and individual data points represent biological replicates. Statistical analyses were performed using unpaired t-test. Ns, not significant.

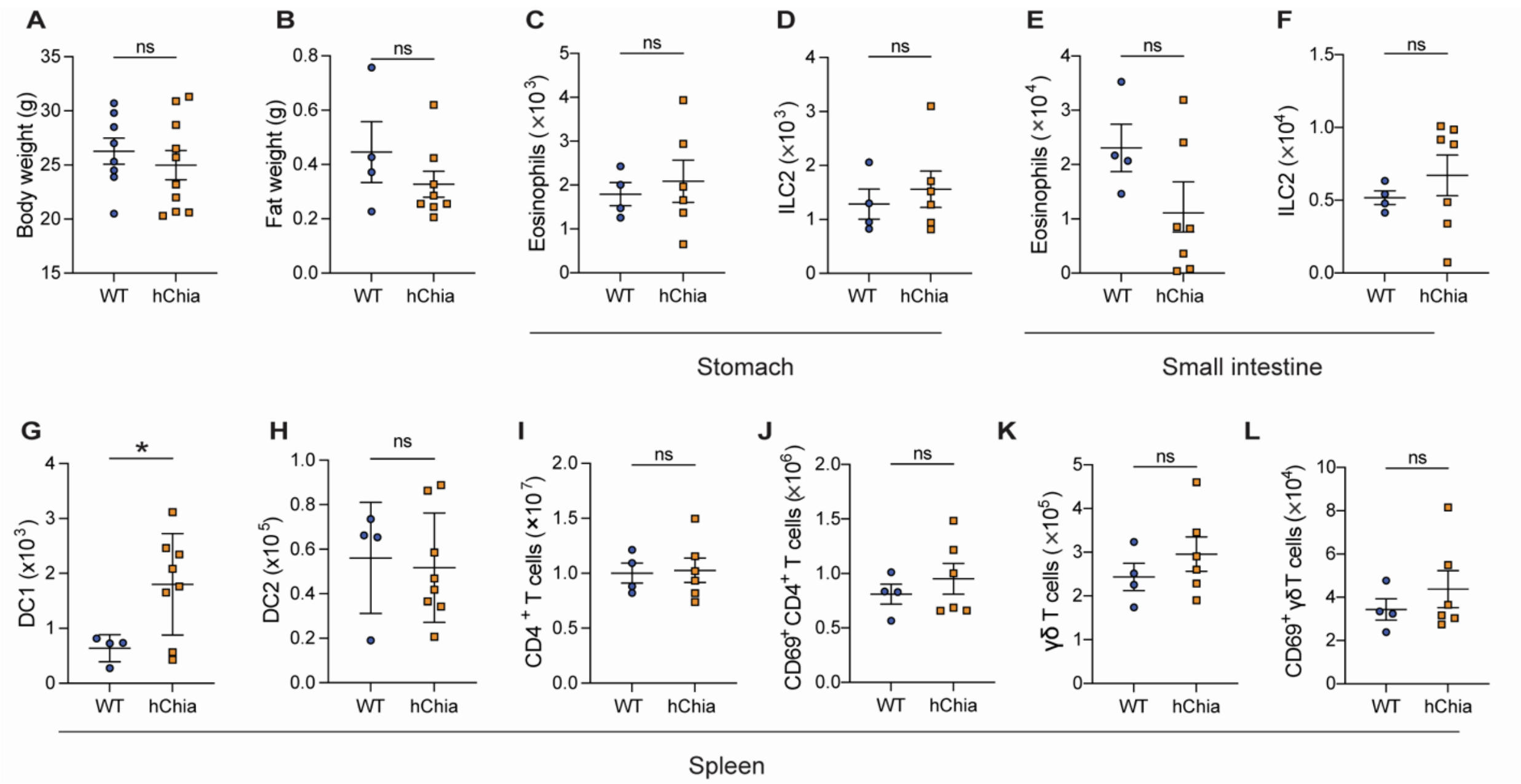

Figure S5. Steady-state characterization of WT and hChia mice

(A-L), Body weight (A), fat weight (B), stomach eosinophils (C), stomach group 2 innate lymphoid cells (ILC2s; D), small intestine eosinophils (E), small intestine ILC2s (F), total spleen DC1 (G), DC2 (H), spleen $CD4^+$ T cells (I), spleen $CD69^+CD4^+$ T cells (J), spleen γδ T cells (K), and spleen $CD69^+$ γδ T cells (L) in indicated mice at steady-state. Data are presented as mean ± SEM and are pooled from at least 2 independent experiments; individual data points represent biological replicates. Statistical analyses were performed using unpaired t-test. *$p < 0.05$; ns, not statistically significant.

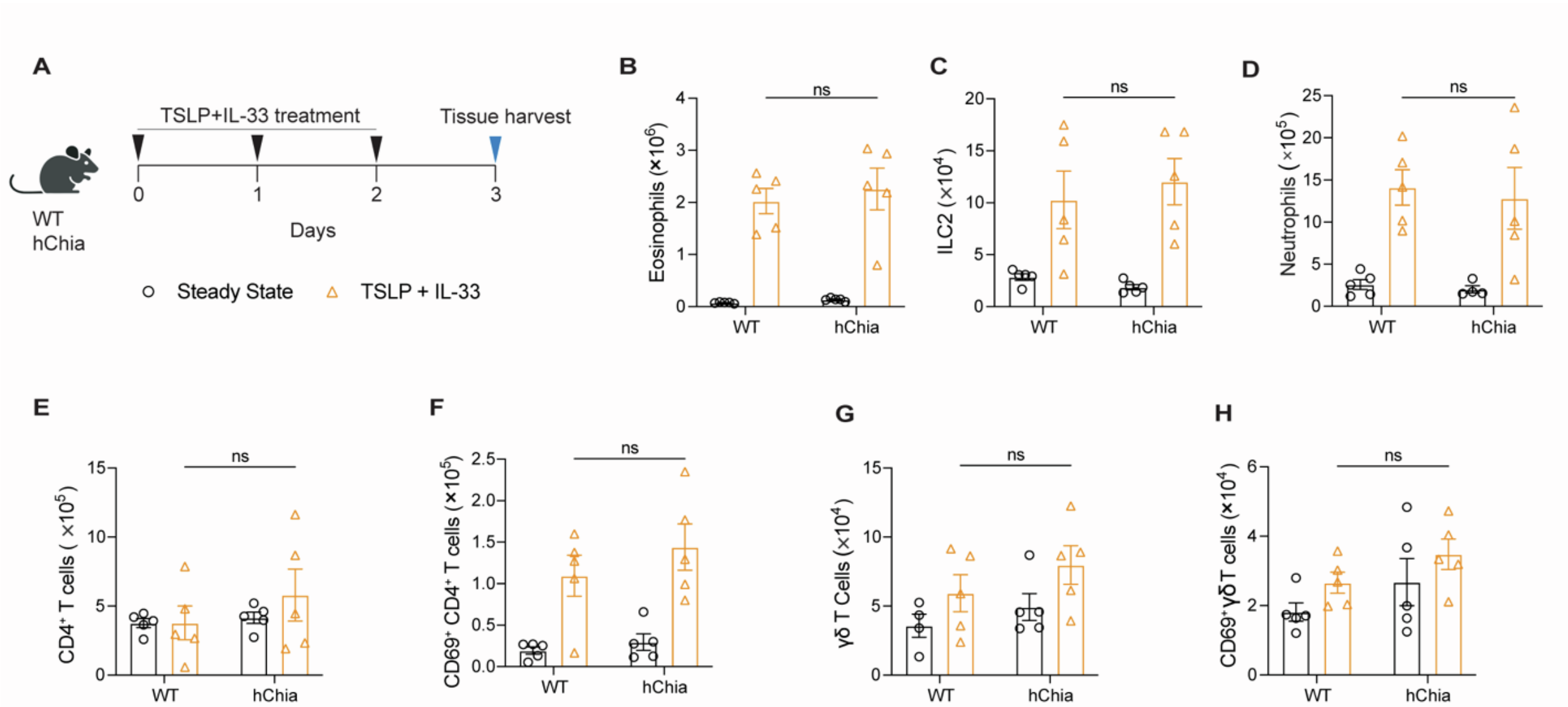

Figure S6. Lung immune responses to TSLP + IL-33 treatment in WT and hChia mice

(A), WT and hChia mice were treated intranasally with TSLP + IL-33 on days 0, 1, and 2, and tissues were harvested on day 3. (B-H) Quantification of eosinophils (B), ILC2s (C), neutrophils (D), $CD4^+$ T cells (E), $CD69^+$ $CD4^+$ T cells (F), γδ T cells (G), and $CD69^+$ γδ T cells (H) in lung tissue from steady-state and TSLP + IL-33-treated mice. Data are presented as mean ± SEM, and individual data points represent biological replicates. Statistical analyses were performed using two-way ANOVA with Sidak's multiple-comparison test. Ns, not statistically significant.

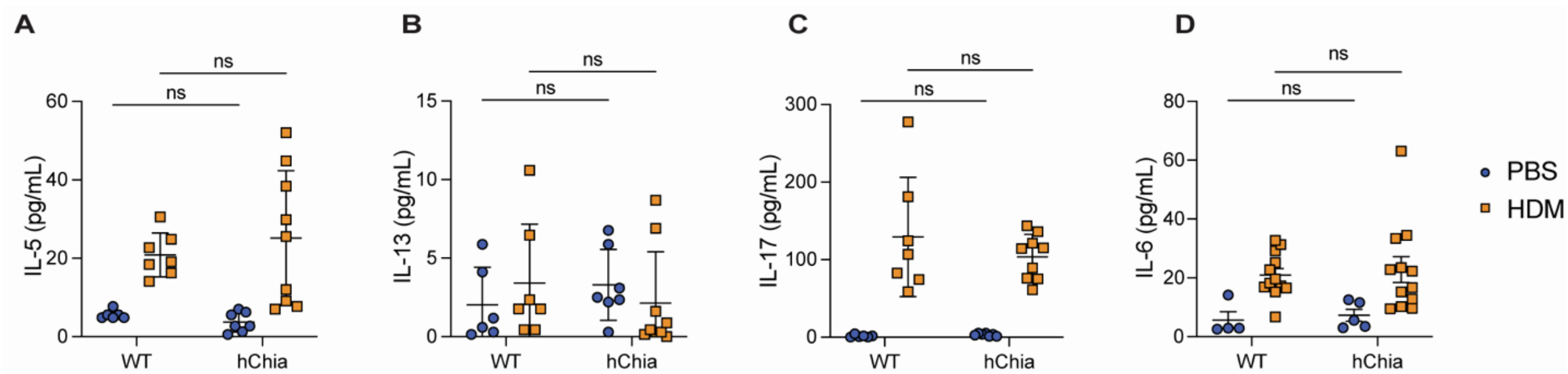

Figure S7. BAL fluid cytokine analysis following HDM challenge in WT and hChia mice

(A-D), BAL fluid levels of IL-5 (A), IL-13 (B), IL-17 (C) and IL-6 (D) measured by ELISA following HDM challenge in WT and hChia mice. Data are presented as mean ± SEM, and individual data points represent biological replicates. Statistical analyses were performed using two-way ANOVA with Sidak's multiple-comparison test. Ns, not statistically significant.

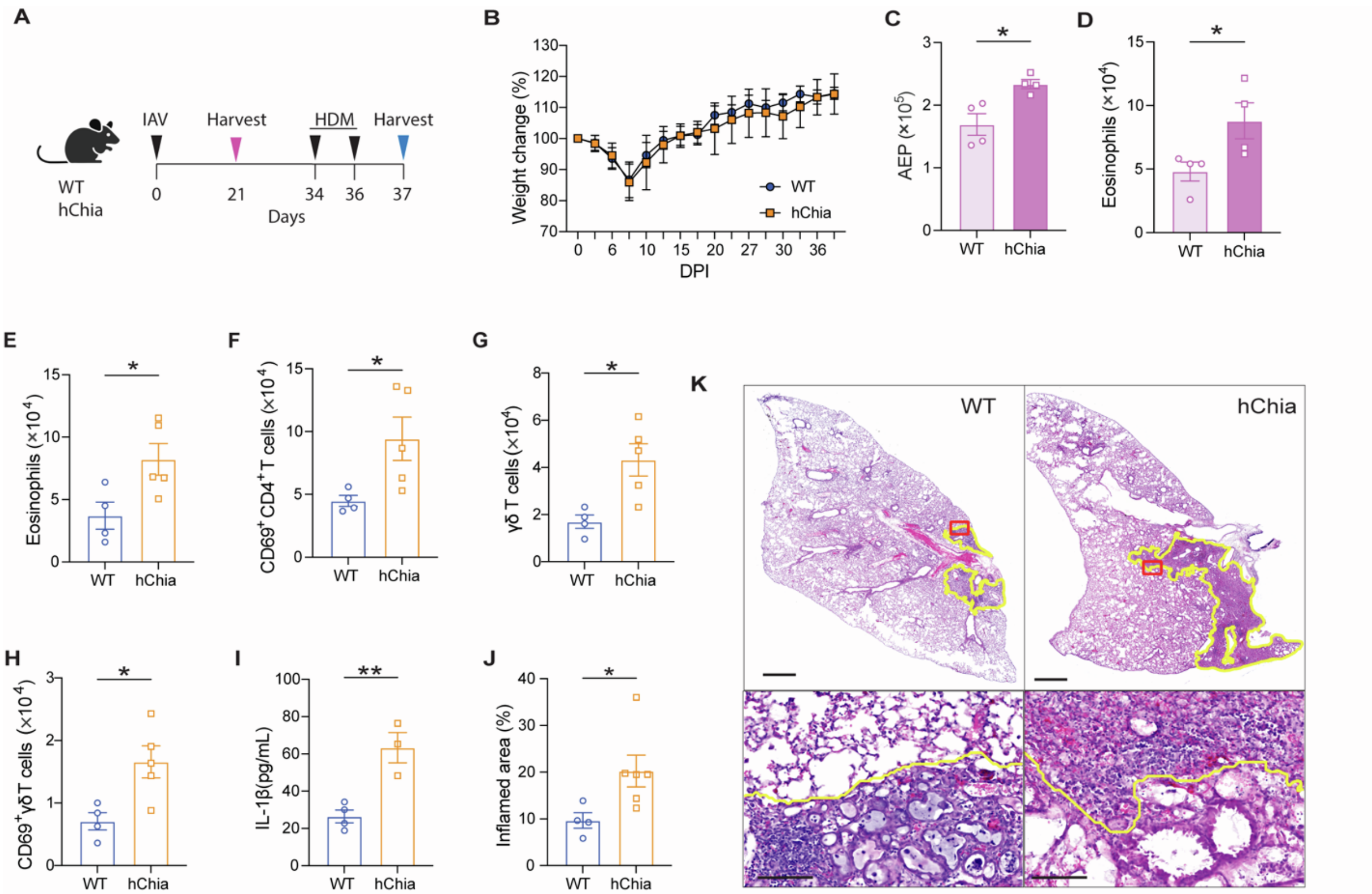

Figure S8. HDM-driven exacerbation of respiratory viral lung disease

(A), Influenza A virus (IAV) inoculation was followed by house dust mite (HDM) challenge in WT and hChia mice as indicated. (B), Body weights of WT and hChia mice following IAV and HDM challenge, shown as percentage of initial weight (n = 6 for WT group and n = 8 for hChia group). (C-D), Flow cytometry quantification of airway epithelial progenitor (AEP) cells (C) and eosinophils (D) in the lungs of WT and hChia mice 21 days post-IAV infection (dpi). (E-H), Eosinophils (E), $CD69^+CD4^+$ T cells (F), γδ T cells (G) and $CD69^+$γδ T cells (H) in the lungs of WT and hChia mice at 37 dpi (1 day after final HDM challenge). (I), IL-1β concentrations in BAL fluid. (J-K) Quantification (J) and representative H&E-stained sections indicating inflamed lung area (K; yellow outline) in mice following IAV and HDM challenge. Scale bars, 1 mm (top) and 100 μm (bottom). Data are presented as mean ± SEM and are pooled from at least 2 independent experiments; individual data points represent biological replicates. Statistical analyses were performed by unpaired t-test (C-J). *$p < 0.05$, **$p < 0.01$.

Table S1. Characteristics of critically ill participants who underwent bronchoalveolar lavage sampling

**Sample Characteristics**	
**Demographics**	
Participants (n)	39
Age (median, sd)	56 ±14
Body mass index (median, sd)	33 ± 10
Sex	
Male (n, %)	24 (62%)
Female (n, %)	15 (38%)
Race	
White (n, %)	23 (59%)
Black (n, %)	14 (36%)
American Indian (n, %)	2 (5%)
**Clinical diagnosis**	
Pneumonia (n, %)	39 (100%)
Respiratory infection (n, %)	25 (64%)
Viral pneumonia (n, %)	15 (38%)
Bacterial pneumonia (n, %)	14 (36%)
**Degree of hypoxemia**[1]	
Minimal (n, %)	1 (3%)
Mild (n, %)	10 (26%)
Moderate (n, %)	21 (54%)
Severe (n, %)	7 (18%)
**Comorbidity history**	
Chronic obstructive pulmonary disease	6 (15%)
Obstructive sleep apnea	9 (23%)
Asthma	4 (10%)
Heart failure	10 (26%)
Chronic kidney disease	9 (23%)
Malignancy	8 (21%)
HIV	6 (15%)
Cirrhosis	4 (10%)

[1] Severity of hypoxemia is determined via the minimum PaO_2 to FiO_2 ratio or equivalent SpO_2 to FiO_2 ratio in the first seven days after development of respiratory failure.

Minimal: PaO2:FiO2 > 300mmHg or SpO2:FiO2 > 315mmHg

Mild: PaO2:FiO2 > 200mmHg and ≤ 300mmHg or SpO2:FiO2 > 235mmHg and ≤ 315mmHg

Moderate: PaO2:FiO2 > 100mmHg and ≤ 200mmHg or SpO2:FiO2 > 148mmHg and ≤ 235mmHg

Severe: PaO2:FiO2 ≤ 100mmHg or SpO2:FiO2 ≤ 148mmHg

Table S2. *CHIA* and *CHIT1* genotypes of patients in this study

Sample number	CHIA genotype	CHIT1 genotype	Genotype score
1	hChia/mouse like	WT/dup	2
2	hChia/hChia	WT/dup	3
3	hChia/mouse like	WT/dup	2
4	hChia/mouse like	WT/dup	2
5	hChia/hChia	WT/WT	1
6	hChia/hChia	WT/WT	1
7	N/A	N/A	N/A
8	N/A	N/A	N/A
9	hChia/hChia	WT/dup	3
10	hChia/hChia	WT/dup	3
11	hChia/hChia	WT/WT	1
12	hChia/mouse like	WT/WT	0
13	N/A	N/A	N/A
14	hChia/hChia	dup/dup	4
15	hChia/hChia	WT/WT	1
16	hChia/hChia	WT/WT	1
17	N/A	N/A	N/A
18	hChia/hChia	WT/WT	1
19	hChia/hChia	WT/dup	3
20	N/A	N/A	N/A
21	hChia/hChia	WT/WT	1
22	hChia/hChia	WT/WT	1
23	hChia/hChia	WT/WT	1
24	hChia/hChia	WT/WT	1
25	hChia/hChia	WT/dup	3
26	hChia/mouse like	WT/WT	0
27	hChia/hChia	WT/WT	1
28	hChia/mouse like	WT/WT	0
29	hChia/hChia	WT/WT	1
30	hChia/hChia	WT/dup	3
31	hChia/mouse like	WT/WT	0
32	hChia/hChia	WT/WT	1
33	hChia/mouse like	WT/dup	2
34	hChia/hChia	WT/WT	1
35	hChia/hChia	WT/WT	1
36	hChia/hChia	WT/WT	1
37	N/A	N/A	N/A
38	hChia/mouse like	WT/WT	0
39	hChia/hChia	WT/WT	1
40	hChia/hChia	WT/WT	1
41	hChia/hChia	WT/dup	3
